# The overlooked conservation values of saline lakes

**DOI:** 10.1038/s41467-026-76684-0

**Published:** 2026-09-03

**Authors:** Alfred Burian, Ramesh Wilson, Pavel Kratina, Örs Ábrám, Ekaterina Afonina, Javier Alcocer, María Belén Alfonso, Tania Anderson, Elena Anufriieva, Nurgul Balci, Luciana Gomes Barbosa, Bonnie K. Baxter, Balzhit Bazarova, Yuri Bazhenov, Michael Beckmann, Emil Boros, Svetlana Borzenko, Enrique H. Bucher, Antonio Camacho, Emilio O. Casamayor, Xiaobo Chao, Peri Coleman, María Florencia Colla, Bindy Datson, Santiago Echaniz, Maria Eugenia Farias, Tadesse Fetahi, Carlos M. García, Larisa Golovatyuk, Peter Hudson, Katia Hueso-Kortekaas, Jill Johnston, Bakhtiyor K. Karimov, Irina Kalioujnaia, Anastasia Komova, Lothar Krienitz, Seema Kulshreshtha, Ron Larson, Oksana Lipka, Evgeniya Matyugina, Quentin Mauvisseau, John Melack, Keely Mills, Daniel Morant, Edmundo Moreno, Zorigto Namsaraev, Aharon Oren, Christopher Rogers, David B. Ryves, Marta I. Sánchez, Alba Camacho-Santamans, Anna C. Santamans, Michael Schagerl, Elena Selivanova, Nickolai Shadrin, Somayeh Sima, Natalya Tashlykova, Katrin Teubner, Gazhit Tsybekmitova, Brian Timms, Svetlana Ulanova, Nicolás E. Vidal Quini, Alicia Vignatti, Wayne A. Wurtsbaugh, Charitos Zapitis, Thomas Zechmeister, Can Zhang, Egor Zadereev

**Affiliations:** 1 Department of Computational Landscape Ecology, Helmholtz Centre for Environmental Research – UFZ, Leipzig, Germany; 2 Ecology Department, Lurio University, Nampula, Mozambique; 3 Department of Biology, University of Oxford, Oxford, UK; 4 School of Biological and Behavioural Sciences, Queen Mary University of London, London, UK; 5 Doctoral School of Natural Sciences, Hungarian University of Agriculture and Life Sciences, Gödöllő, Hungary; 6 Institute of Natural Resources, Ecology and Cryology SB RAS, Chita, Russia; 7 FES Iztacala, GILT, Universidad Nacional Autónoma de México, Tlalnepantla, Estado de México Mexico; 8 Research Institute for Applied Mechanics (RIAM), Kyushu University, Kasuga, Fukuoka Prefecture Japan; 9 FitzPatrick Institute of African Ornithology, University of Cape Town, Cape Town, South Africa; 10 Kovalevsky Institute of Biology of the Southern Seas, RAS, Sevastopol, Russia; 11 Department of Geological Engineering, Geomicrobiology & Biogeochemistry Laboratory, Istanbul Technical University, Istanbul, Turkey; 12 Department of Geology, University of Georgia, Athens, GA USA; 13 Departament of Phytotechnology and Environmental Sciences, Federal University of Paraiba, João Pessoa, Brazil; 14 Great Salt Lake Institute, Westminster University, Salt Lake City, USA; 15 Faculty for Environment and Natural Sciences, Brandenburg University of Technology Cottbus-Senftenberg, Cottbus, Germany; 16 Department of Geography and Natural Sciences, Department of Aquatic Environmental Sciences, Ludovika University of Public Service, Budapest, Hungary; 17 National University of Cordoba, Cordoba, Argentina; 18 Cavanilles Institute of Biodiversity and Evolutionary Biology, University of Valencia, Paterna, Valencia, Spain; 19 Department of Ecology and Complexity, Center of Advanced Studies of Blanes, CEAB-CSIC, Blanes, Spain; 20 National Center for Computational Hydroscience and Engineering, The University of Mississippi, Oxford, USA; 21 Delta Environmental Consulting, St Kilda, Australia; 22 Laboratorio de Humedales, Centro Regional de Energía y Ambiente para el Desarrollo Sustentable (CREAS-CONICET-UNCA), San Fernando del Valle de Catamarca, Catamarca, Argentina; 23 Actis Environmental Services, Actis Consulting Pty Ltd, Perth, Australia; 24 Department of Biological Sciences, National University of La Pampa, Santa Rosa, La Pampa Argentina; 25 PUNABIO S.A. Campus USP-T, San Pablo, Argentina; 26 Department of Zoological Sciences, Addis Ababa University, Addis Ababa, Ethiopia; 27 Universidad de Cádiz, Cadiz, Spain; 28 Papanin Institute for Biology of Inland Waters, Borok, Russia; 29 Earth & Biological Sciences, South Australian Museum, Adelaide, South Australia Australia; 30 Department of Mechanical Engineering, ICAI School of Engineering, Comillas Pontifical University, Madrid, Spain; 31 Department of Population and Public Health Sciences, University of Southern California, Los Angeles, USA; 32 Ecology and Water resources management, National Research University “Tashkent institute of irrigation and agricultural mechanisation engineers”, Tashkent, Uzbekistan; 33 Faculty of Geography, Lomonosov Moscow State University, Moscow, Russia; 34 Kurchatov centre for genome research, NRC Kurchatov Institute, Moscow, Russia; 35 Plankton and Microbial Ecology, Leibniz Institute of Freshwater Ecology and Inland Fisheries, Stechlin, Germany; 36 Department of Zoology, Govt Shakambhar PG College, Jaipur, Rajasthan India; 37 US Fish and Wildlife Service, Klamath Falls, USA; 38 Yu.A. Izrael Institute of Global Climate and Ecology, Moscow, Russia; 39 Natural History Museum, University of Oslo, Oslo, Norway; 40 University of California, Santa Barbara, USA; 41 British Geological Survey, Keyworth, UK; 42 Professor at Universidad Nacional del Altiplano, Puno, Peru; 43 The Edmond J. Safra Campus, The Hebrew University of Jerusalem, Jerusalem, Israel; 44 Scenic Rivers & Watershed Research Laboratory, GRDA, Tahlequah, OK USA; 45 Department of Geography and Environment, Loughborough University, Loughborough, UK; 46 Conservation Biology and Global Change, Doñana Biological Station, Sevilla, Spain; 47 Departament de Biologia Evolutiva, Ecologia i Ciències Ambientals, University of Barcelona, Barcelona, Catalonia Spain; 48 Department of Functional and Evolutionary Ecology, University of Vienna, Vienna, Austria; 49 Institute for Cellular and Intracellular Symbiosis, Orenburg Federal Research Center, UB RAS, Orenburg, Russia; 50 Faculty of Civil and Environmental Engineering, Tarbiat Modares University, Tehran, Tehran Iran; 51 Independent Researcher, Morisset, New South Wales Australia; 52 The Institute of Complex Research of Arid Areas, Republic of Kalmykia, Elista, Russia; 53 Departamento de Geografia y Turismo, Universidad Nacional del Sur, Bahía Blanca, Argentina; 54 Watershed Sciences & Ecology Center, Utah State University, Logan, UT USA; 55 Chief Scientist Directorate, Natural England, Nottingham, UK; 56 Department of Agriculture, Nature Conservation and Environmental Protection, Biological Station Lake Neusiedl, Government of Burgenland, Austria, llmitz, Austria; 57 School of Geography and Ocean Science, Nanjing University, Nanjing, China; 58 Institute of Biophysics, Federal Research Center “Krasnoyarsk Science Center SB RAS”, Krasnoyarsk, Russia; 59 Siberian Federal University, Krasnoyarsk, Russia

**Keywords:** Conservation biology, Ecosystem services, Biodiversity

## Abstract

Saline lakes are hypersensitive to changes in their water balance and therefore show amplified responses to climatic and land-use changes in their catchment. Despite often dramatic ecological impacts, saline lakes rank low on policy agendas as they are assumed to support few ecosystem services and low levels of biodiversity. Here, we challenge this view and evaluate ecosystem services and threatened species in 85 saline lakes distributed across the globe. We show that saline lakes support, additionally to threatened aquatic biota, a diverse range of red-listed terrestrial species that contribute together with a large beta diversity to their conservation value. Further, our results highlight that saline lakes provide a number of culturally and economically important ecosystem services but several of them are ‘hidden’ and difficult to quantify. We conclude our analysis with best-practice recommendations for sustainable management of saline lakes. Their local adaptation and implementation will be key for safeguarding biodiversity and ecosystem services of these valuable and highly sensitive ecosystems.

## Abstract (web summary)

Saline lakes are highly threatened ecosystems, yet they receive little attention from both the conservation community and the general public. This study demonstrates why these systems are important biodiversity refuges and highlight the ecosystem services they provide.

## Introduction

Saline lakes are a diverse group of ecosystems that account for over 45% of the global lake water volume and 23% of global lake surface area^1^. Their high environmental diversity is partly rooted in the wide spectrum of salinities, ranging from brackish ecosystems (saline lakes are here defined^2^ as those with a salinity of > 1 g L^−1^) to saturated brines with ten times higher salt concentrations than oceans^3^. The often-unique character of individual lakes further emerges from differences in ion composition, extent of lake level variation, and desiccation frequency, which can be key factors shaping lake communities^4–6^. The vast majority of saline lakes, nonetheless, share a common denominator – a high sensitivity to climate and land-use changes in their catchment^7^ that turns them into sentinels of global and local environmental change^8^.

The main reason for their sensitivity, which causes saline lakes to rank among the most threatened ecosystem types worldwide, is that nearly all saline lakes are terminal systems with no surface outflow^9^. Hence, lake water levels depend on a sensitive balance between water inflows and evaporation^10^. This sensitivity does not only drive large natural inter- and intra-annual variations in lake water levels, which can even cause seasonal desiccation, but also exacerbates the effects of climatic changes and land-use intensification. Prominent recent examples of the consequences are dramatic drops in lake levels in the Aral Sea, the Great Salt Lake, Urmia Lake and the Dead Sea^11–14^ or flooding of East African soda lakes due to increased rainfall^15^.

Shifts in lake levels are accompanied by strong salinity changes, which have critical physiological implications and often lead to disruptions of aquatic communities^16–18^. Nonetheless, saline lakes are frequently marginalised and rank low on policy agendas, as they are believed to support few ecosystem services and low levels of biodiversity^4,19^. This perception is partly rooted in a focus on drinking water and fish yields, which are key ecosystem services in freshwater ecosystems but less relevant in saline lakes. By contrast, other uses such as salt extraction^20^, therapeutic bathing^21^, commercial harvests of invertebrates (e.g., *Artemia* harvest is worth over $70 million dollars year^−1^ in the Great Salt Lake^22^), and a suite of fundamental supporting ecosystem services are commonly overlooked.

The preconception that saline lakes support low levels of biodiversity refers to the simple metazoan food webs typically found in these environments, particularly at higher salinities^23,24^. This low diversity of aquatic metazoans, however, is often contrasted by diverse microbial communities^25,26^ that constitute a valuable natural capital with the potential to spark industrial innovations^27^. Moreover, the accumulation of nutrients in the basin of saline lakes can lead to extremely high primary production rates^28^. The resulting high algae, plant, and invertebrate biomass can be an important subsidy fuelling terrestrial food webs and can turn saline lakes into sanctuaries for many terrestrial species. Especially many migrating bird species critically rely on saline lakes as they use them as feeding stops on migration routes in otherwise barren drylands^29–31^.

**Fig. 1:**
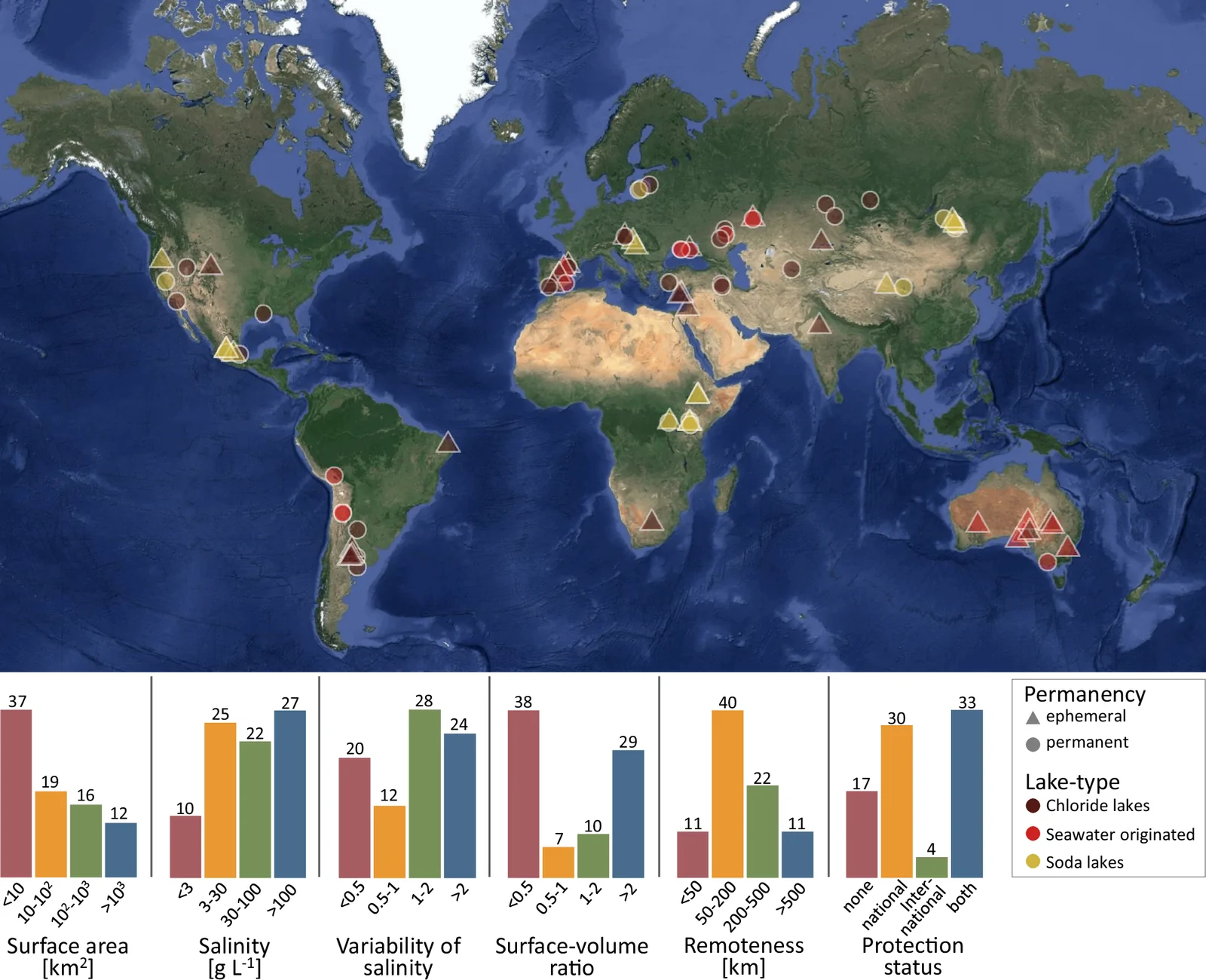
Location and characteristics of the 85 saline lakes included in this study. A global map in the top panel displays locations, ion-types, and permanency of included lakes. Histograms in the bottom panels present the distribution of six other environmental and management characteristics that were included in addition to ion-type and permanency as predictors in our regression models. The basemap originates from Google Maps Platform.

However, neither the provision of ecosystem services nor the conservation value of saline lakes have been systematically evaluated. Hence, we assess here both ecosystem services and threatened species from 85 saline lakes distributed across the globe (Fig. 1 and Supplementary Data 1, 2). Our aim is to identify, based on expert surveys that integrated scientific, conservation, and local knowledge, the key environmental drivers of red-listed species occurrence and ecosystem service provisioning. However, the number of supported threatened species and ecosystem services are, despite their common use, only partially reflecting the value of ecosystems, and hence we highlight their limitation in the context of saline lakes. We complement these analyses with a replicated expert consultation to synthesise major management challenges and best-practice recommendations for safeguarding biodiversity and service provisioning in saline lake ecosystems.

## Results and discussion

### Threatened species and the conservation value of saline lakes

**Fig. 2:**
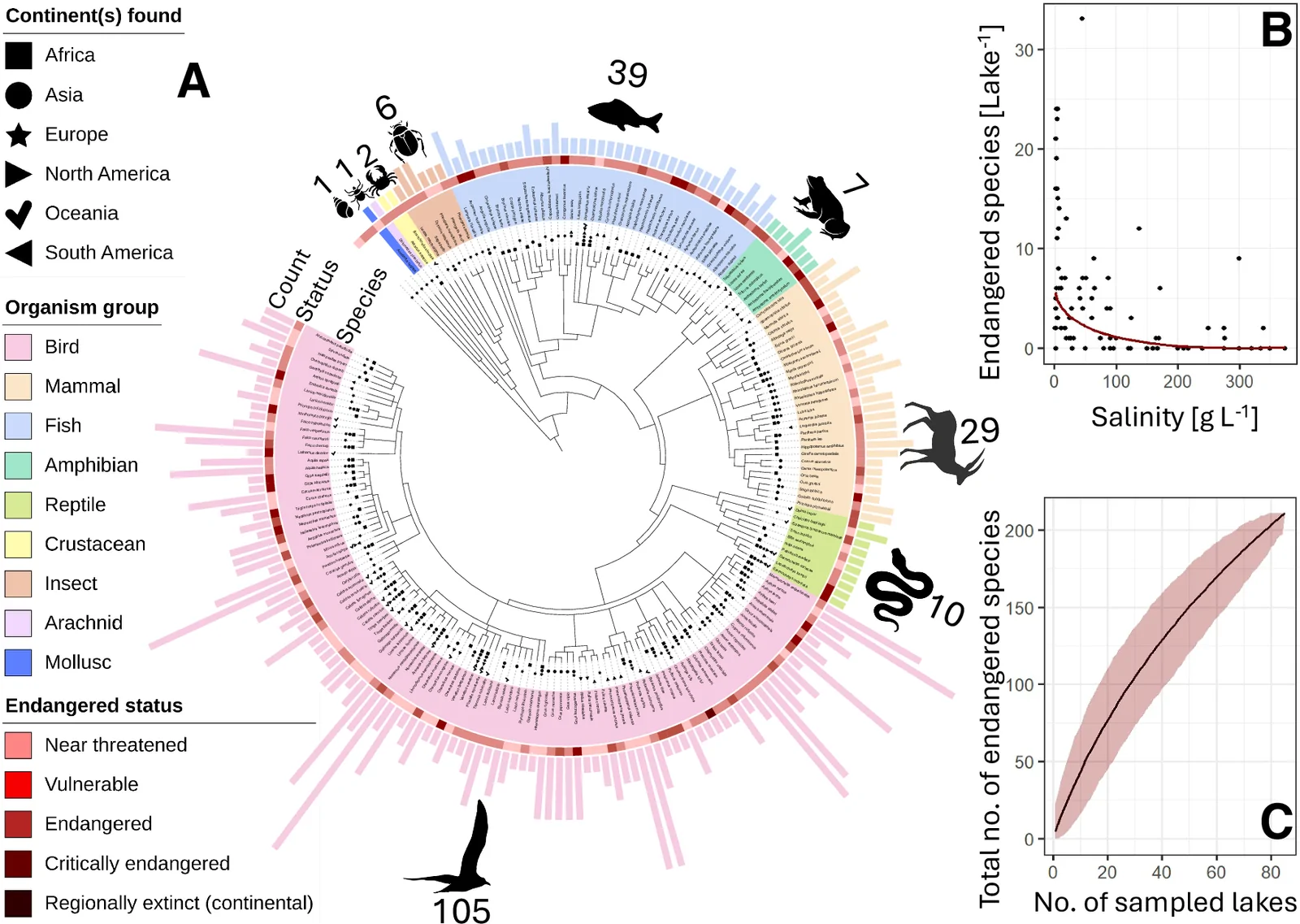
Threatened species found in saline lakes and the key drivers of their occurrence. **A** Phylogenetic tree showing the taxa that have been reported to live in or to be strongly dependent on saline lakes. Their taxonomic affiliation, their geographical occurrence and their conservation status are also displayed. Lengths of bars show species counts in the dataset. **B** The impact of salinity, the most important predictor of threatened species richness per lake. **C** The total number of endangered species as a function of lake sample size (i.e., the number of considered lakes). The solid line represents the mean of 5000 random draws from the lake database of this study. The red-shaded area represents the 95% confidence interval band. The lack of a strongly saturating response curve demonstrates the often very distinctive species composition of individual lakes, resulting in high beta-diversity of saline inland waters. Attribution links for icon-artwork are tick icon https://www.flaticon.com/authors/vector-stall for tick, https://www.flaticon.com/authors/freepik for the crab and for the bird, for the snail https://www.flaticon.com/authors/mayor-icons for the snail, https://www.flaticon.com/authors/park-jisun for the beetle, https://www.flaticon.com/authors/planbstudio for the fish, https://www.flaticon.com/authors/anditii-creative for the frog, https://www.flaticon.com/authors/delwar018 for the antelope and https://www.flaticon.com/authors/unknown-depths for the snake.

In total, we recorded 200 species listed as threatened or near threatened by the IUCN that occurred across 27 soda, 40 inland sulphate and chloride and 18 coastal seawater lakes with a salinity range of 1 g L^−1^ to 375 g L^−1^ (Fig. 2 and Supplementary Data 3, 4; further referred to simply as threatened species). On average, a lake hosted five threatened species (median of three), a number that accounts for all species that critically rely on the existence of the lake. Hence, the constructed tree of endangered life that is supported by saline lakes (Fig. 2) encompasses aquatic but also many terrestrial species. Birds were prominently represented and accounted for 53% of all threatened species, which reflects the importance of saline lakes as feeding, nesting, and stopover sites^29,30^. Therefore, evaluations of saline lakes based solely on their aquatic fauna would lead to considerable underestimation of their importance for preserving red-listed species.

The high proportion of birds and other vertebrates in our threatened species list emphasises the relevance of saline lakes for these groups. However, the global red listing of threatened species is a major endeavour that is far more advanced for fish, birds, and mammals than for invertebrates^32^. This underrepresentation of invertebrates may result in an undervaluation of the conservation value of inland waters^33^ and specifically saline lakes^34^. We recorded, for example, almost 500 different dominant plankton species across our investigated saline lakes. None of these species is listed as threatened, although some, such as *Artemia monica* and *A. persimillis*, are endemic to highly stressed ecosystems^35,36^. Moreover, saline lakes host highly productive and taxonomically often very distinct microbial communities^37^ that remain unaccounted for and thereby highlight the challenges that emerge from systematic bias in red lists for the protection of saline lakes.

The number of red-listed species supported per saline lake declined with salinity (*p* < 0.001, Fig. 2B and Supplementary Table S1). Salinity was the only predictor variable in the model with the lowest AIC, and its negative impact on the richness of endangered species per lake accords with the reduction of aquatic and terrestrial biodiversity in highly saline lakes^38–40^. However, diversity should not only be evaluated at the species level, but according to the Convention of Biological Diversity, genetic and ecosystem diversity should be equally considered^41^. We found that highly saline lakes often harbour a unique set of endangered species and show large differences among lakes. For example, Lake Krasnovishnevoye (Russia), Lake Urmia (Iran) and Seagull Lake (Australia) all have average salinities above 100 g L^−1^, but each supported at least two endangered species that were found in no other lake of our dataset. This high diversity at the ecosystem level was also evident from rarefaction curves depicting the relationship between the total count of endangered species and the number of considered saline lakes (Fig. 2C). This relationship showed little signs of saturation, although we covered 85 lakes in our analysis, highlighting the often unique character of individual saline lakes.

Genetic diversity was not a specific focus in our study. Nonetheless, it is important to consider the genetic impact of the extreme and often variable environments in saline lakes. The resulting selection pressure has favoured adaptive and stress-tolerant communities, which contain unique and valuable genes facilitating extreme physiological adaptations^42^. Conserving saline lakes, hence, helps to maintain gene reservoirs, which support the planet’s capacity to adapt to local and global environmental changes. Therefore, evaluating the conservation value of saline lakes requires accounting for (i) strong aquatic-terrestrial coupling that largely extends the number of threatened species that depend on saline lakes, (ii) the large diversity at the ecosystem level, and (iii) the unique gene sets that are only encountered in saline lakes.

### Drivers of ecosystem service provisioning

**Fig. 3:**
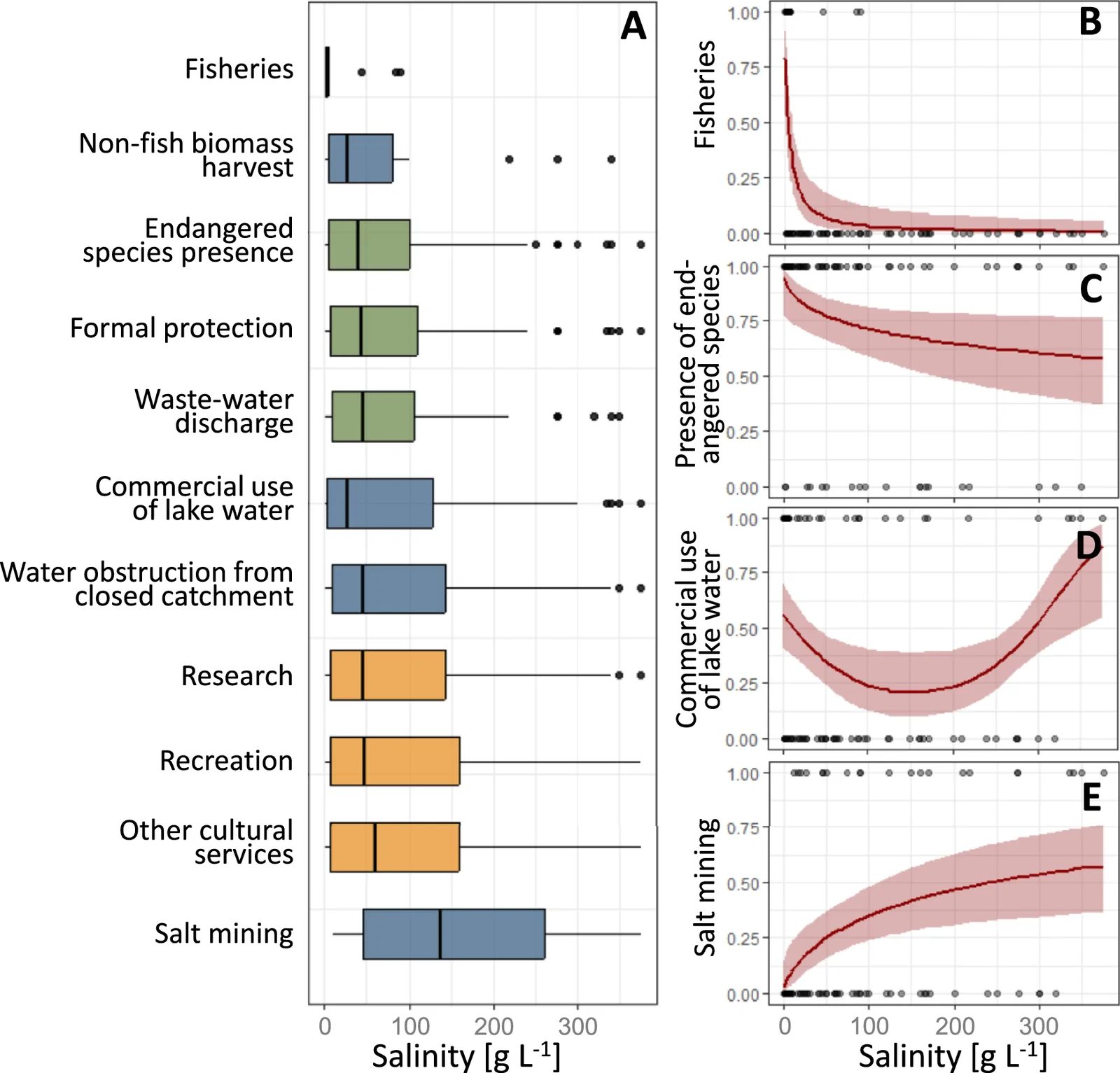
The provisioning of individual ecosystem service and their relationship with salinity. **A** The distribution of average salinity values of lakes that provide a given service. Colours reflect provisioning (blue), supporting and regulating (green) and cultural (yellow) ecosystem services. The presence of endangered species and the formal protection of a lake by national law or international agreements (RAMSAR) are used as indicators for biodiversity, and, hence supporting ecosystem services. **B**–**E** The probability of provisioning individual ecosystem service was modelled as a function of salinity using logistic regressions. Displayed are all significant results (*p* < 0.05). Red lines represent regression predictions, and red bands indicate 95% confidence interval bands. Boxplots in A display median, interquartile range (coloured area), range of data without outlayers (whiskers) and out-layers (points; distance to quartile is larger than 1.5 times the interquartile range). Equations of regression predictions are displayed in Supplementary Table S1.

Our survey considered 11 key categories of ecosystem services (Fig. 3). Cultural services were most frequently listed for individual lakes. For example, the support of research (i.e., more than 20 papers in Web of Science targeting a lake) or use for tourism and recreation were recorded for 88.4 and 83.7% of all lakes, respectively. Provisioning and regulating ecosystem services were individually less common but more diversified, including fisheries (19.8%), non-fish biomass harvest (24.4%), salt mining (25.6%) or the use for waste-water discharge (41.9%; wastewater was untreated in most cases). Additionally, saline lakes provide a number of essential supporting and regulating ecosystem services. However, these services were not included in our quantitative analysis as they are difficult to measure, and we instead evaluate their importance in the section ‘truly hidden services of saline lakes’ below.

**Fig. 4:**
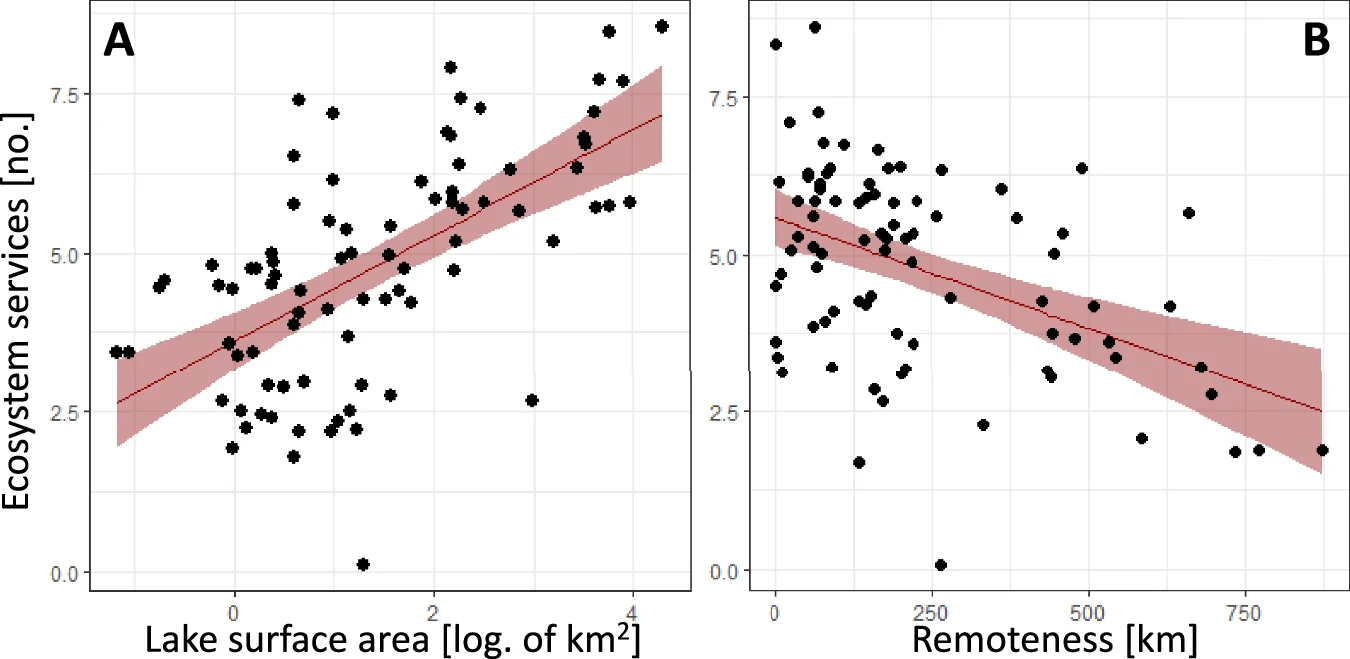
The relationship between the number of ecosystem services provided by saline lakes and lake surface area (A), and remoteness (B), the two predictors that were included in the most parsimonious model. The y-axes show the sum of the effect of the predictor and model residuals, and, hence, the confounding effect of the other predictor has been accounted for in the graphical display. Red lines show model predictions of regression analyses, and red areas reflect 95% confidence interval bands. Equations of regression predictions are displayed in Supplementary Table S1.

On average, we recorded five different ecosystem services per lake (formal protection and the presence of endangered species were included as indicators of supporting ecosystem services, see Methods). Lake surface area and remoteness represented key factors influencing the number of ecosystem services provided per lake (Fig. 4 and Supplementary Table S1). The positive effect of lake surface area likely emerges from the larger total biomass supported by larger lakes. The resulting larger natural populations can enhance the touristic value as in the case of flamingos^43^ and ease the commercial exploitation of fish and invertebrates. In contrast, the lower provision of ecosystem services in remote lakes likely reflects their restricted accessibility. This low accessibility limits direct resource exploitation and cultural activities, but should not be misinterpreted as low potential of such lakes.

Contrary to our initial expectations, salinity did not significantly affect the number of ecosystem services provided per lake. This overall neutral effect was the result of both positive and negative salinity impacts on individual ecosystem services (Fig. 3). For example, fisheries markedly declined with average salinity (Supplementary Table S1), as both fish abundance and diversity decrease at salinities above 20–30 g L^−1 ^^44^. Notably, average harvest rates in fished lakes were very high and with 2.2 t km^−2^ yr^−1^ (Table 1) almost matching the highest rates recorded for freshwater lakes (estimated at 2.5 t km^−2^ yr^−1 ^^45^. Further, these fish harvests are often distant from marine and freshwater lakes, making the product more valuable for local consumption. Similar to fish harvest and in accordance with our earlier analysis, the probability of hosting endangered species decreased slightly but significantly with salinity (Fig. 3C).

**Table 1:** Summary of quantitatively evaluated ecosystem services of saline lakes

Type of service	Unit	No. of lakes with service/with estimate	Mean	Median	Max	Min	Sign. salinity effect
Commercial fishery	t year^−1^ km^−2^	17/12	2.2	0.5	11.4	0.1	Yes
Wastewater discharge	m^3^ year^−1^ km^−2^	36/13	8.4 10^5^	2.0 10^4^	8.2 10^6^	8.9	No
Use of lake water	m^3^ year^−1^ km^−2^	34/10	5.0 10^5^	8.2 10^5^	3.7 10^6^	3.8	Yes
Water extraction from the catchment	m^3^ year^−1^ km^−2^	51/16	1.4 10^6^	4.6 10^4^	1.7 10^7^	3.8	No
Recreation	visitors year^−1^ km^−2^	72/41	1.1 10^6^	4.1 10^2^	2.5 10^7^	1.4	No
Salt mining	t year^−1^ km^−2^	21/12	4492	1490	19344	5	Yes

Individual services were only provided for a subset of lakes. Quantitative information on ecosystem services was limited for some of these lakes, and thus, quantitative estimates were not available for all lakes (column 3). The last column indicates whether the likelihood of service provisioning was significantly linked to average salinity (see Fig. 3).

In contrast, salt mining was much more frequent at higher salinities (Fig. 3E and Supplementary Table S1) as salinity augments the financial profitability of salt extractions. Nonetheless, evaporation ponds for salt production are sometimes established in lakes with lower salinities^46^, where they can compete with regulating and supporting ecosystem services^47^. The last ecosystem service that was significantly impacted by salinity was the commercial use of lake water, showing a U-shaped pattern (Fig. 3D and Supplementary Table S1). This non-linear response emerged because of the extraction of less saline water for agriculture and the use of highly saline water for industrial purposes.

It is important to emphasise that we list and present the provision of these ecosystem services as a positive element. However, in case of overuse, almost all of the reported ecosystem services can turn into stressors, threatening the functioning of individual lakes (e.g., lithium mining in the Andes^47,48^). When balancing the benefits and conservation implications of different uses, it will be key to consider the natural variability of saline lakes. The often high variability in environmental conditions is also transmitted to the provision of ecosystem services, and hence, precautionary principles should be applied when establishing thresholds for the sustainable use of ecosystem services.

### The truly hidden services of saline lakes

The 11 ecosystem services we considered in our survey represent a comparably large number (only 2% of previous studies on ecosystem services in lakes consider more than seven services^49^). Yet, there is a range of additional services that are inherently difficult to measure and hence have not been included in our quantitative analyses. Nonetheless, these services should play a core consideration when assessing the functional importance of saline lake systems.

First, there are several regulating and supporting ecosystem services linked to the existence of saline lakes. A key consideration in this regard should be the impact of saline lakes on the regional microclimate. For example, Lake Turkana, a large desert lake in the border region between Kenya and Ethiopia, exhibits mean daily evaporation rates of 55 × 10^6 ^m^3^, and strong south-eastern winds transport this water vapour to South Sudan^50^. Impacts on regional microclimate are difficult to estimate, but their magnitude is revealed through ecological disasters such as the partial desiccation of the Aral Sea^12^. Further, the existence of saline lakes prevents the transformation of local winds into dust storms that are rich in salts and other particles. Such storms have been recorded in many instances and can have profound negative consequences for adjacent habitats and for the health of the local human population^12,51^.

Moreover, saline lakes commonly harbour highly active microbial communities that account for some of the highest measured areal primary and bacterial production rates^24,28^. This high productivity transmits to several ecosystem services such as nutrient recycling, gaseous exchange and carbon sequestration^52^. Global contributions of saline lakes to these processes are certainly orders of magnitude lower than in marine systems. However, given their similar total volume^1^ and their high areal rates^24^, they should be comparable to those by freshwater lakes.

A second group of services that should be considered are services that have not yet materialised and which are likewise linked primarily to microbial communities of saline lakes. Examples are high production rates of valuable biochemicals such as beta-carotene or glycerol that could be commercially utilised^53^. However, microbial communities in many saline lakes remain largely under-researched^54^, and we have only scratched the surface in understanding their functional diversity (exemplified by the discovery of hydrocarbon degradation capabilities of microbes in Antarctic saline lakes^55^). Hence, microbial communities in saline lakes should be similar to plant and insect diversity in rainforests, not only valued for their intrinsic value but also for their partly unknown functional diversity.

In summary, the functional importance of saline lakes stems from the currently provided cultural and provisioning services, difficult-to-quantify supporting and regulating services, and their potential to support new services and innovations. The relative importance of these groups of ecosystem services is lake-specific and dependent on personal values and preferences. Hence, it is neither our intention to evaluate the absolute functional value of saline lakes nor to compare their value to those of freshwater and marine systems. However, in contrast to marine and freshwater systems, saline lakes are often perceived as simply a part of their often dry and barren surroundings with little functional importance. Our assessment revealed the many and sometimes hidden ecosystem services saline lakes are providing and therefore refutes this notion.

### Best practice recommendations for securing saline lake ecosystems

As part of our assessment, we asked experts to rank the main environmental and anthropogenic threats to saline lakes (Supplementary Table S1). The results highlight the multiple and likely interacting stressors threatening saline lakes, as climate change (28% of all votes), land use change (24%) and the (over) utilisation of natural resources (22%) were all considered of very similar importance. These threats and sensitivity to environmental change^13^ highlight the need to effectively protect and manage saline lake ecosystems.

**Fig. 5:**
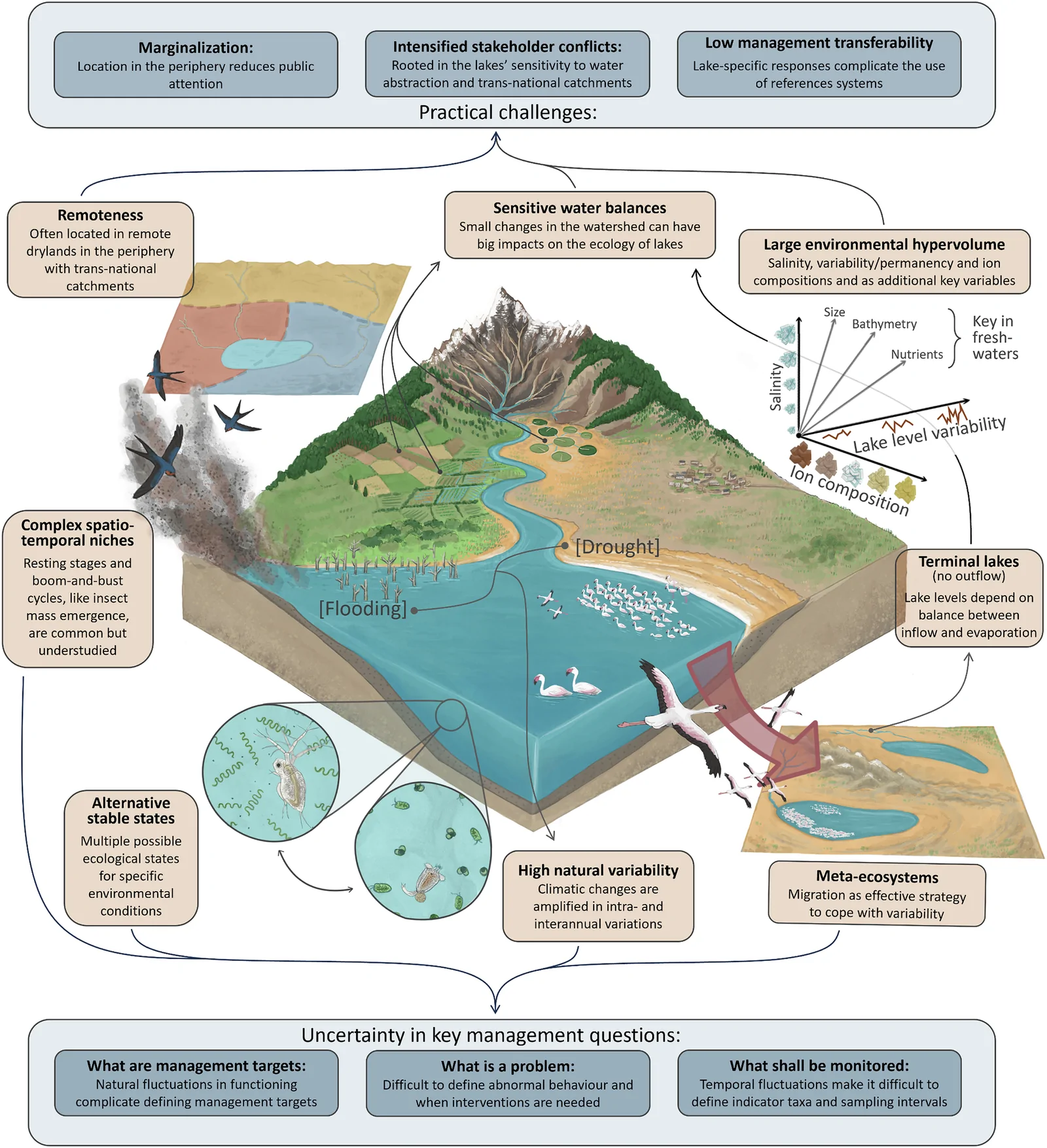
Main environmental and biotic characteristics of saline lakes and the management challenges (blue boxes) they pose. Central are terminal catchments, which is a feature of almost all saline lakes, driving their high natural variability (sometimes complex interactions with groundwater are not illustrated for simplicity). Resulting biotic characteristics and adaptations, such as complex spatio-temporal niches, the existence of alternative states, and the linkage of individual lakes into meta-ecosystems, make it difficult to define key management questions (bottom). Additional features, such as the large environmental diversity of saline lakes and their often transnational catchments, result in practical management challenges (top).

Although saline lakes in some countries and regions have started to be recognised and protected as valuable ecosystems^56^, the large majority of lakes lacks comprehensive management concepts. This lack of long-term planning is partly a result of the marginalisation of saline lakes, but partly also a consequence of the inherent characteristics of saline lake systems^57^ that provide multiple challenges for their management (see Supplementary Note 1 for extended discussion on biomonitoring of saline lakes under the EU Water Framework Directive). We, therefore, in a replicated expert consultation process, evaluated the main characteristics of saline lakes that complicate the identification of management and monitoring targets (Fig. 5). We then synthesised the following five best practice recommendations to overcome these challenges:

1. Catchment-wide management plans: Saline lakes are highly sensitive to hydrological changes in their catchment. Integrated water-management plans are also important for freshwater lakes (e.g., to prevent eutrophication). However, in saline lakes, a failure to account for catchment-wide water balances can lead to the disappearance of entire lake systems and hence catchment wide-water management is of even greater urgency. Key challenges in this regard are to account for (i) groundwater flows, which are poorly understood in most saline lakes but likely of high importance^58^ and (ii) the large natural interannual variability of saline lakes^59^. Interannual variability in, for example, precipitation complicates impact assessments of systematic water withdrawal, which might be under or overrated if overlapping with a period of naturally high or low water-levels, respectively. Hence, long-term monitoring data and appropriate catchment-wide in situ measurements are key requirements for the establishment of sustainable management plans.
2. Accounting for alternative states and strong inter-lake connectivity: The large environmental variability of saline lakes has led to the evolution of coping strategies such as dormancy or migration in many taxa. These coping strategies can lead to boom- and bust-cycles^60^, a strong interconnectedness of individual lakes into meta-ecosystems^61–63^, and the existence of multiple alternative pristine ecological states^64^. Consequently, natural reference conditions encompass often a range of community configurations rather than a single state, as in most freshwater systems, where alternative stable states are largely triggered by anthropogenic stressors. The resulting ambiguity needs to be accounted for when monitoring ecological communities of saline lakes. Monitoring schemes should therefore consider (i) the evaluation of species richness as time-integrated alpha diversity through repeated sampling and (ii) to assess the viability of populations by combined sampling across interconnected lakes.
3. Accounting for time-delayed and strongly lake-specific responses: Environmental heterogeneity is especially strong in saline lakes. As in freshwater systems, size, bathymetry, and nutrient concentrations are important environmental variables differentiating lake ecosystems. However, saline lakes also have large differences in salinity levels, ion composition, and temporal variability (e.g., astatic vs. static water bodies), which function as additional strong selection factors for natural communities^44^. The resulting higher dimensionality of Hutchison’s niche spaces frequently leads to a strongly individual character of saline lakes and a lower transferability of management concepts, which need to be adjusted to lake-specific conditions. Moreover, saline lakes often have delayed responses to management and land-use changes. For example, hydrological changes in the catchment of the Great Salt Lake took 15 years to manifest fully their impact on the lake ecosystem^65^. Likewise, ecological effects can be temporally buffered (e.g., through egg-banks in sediments^66^) until sudden population breakdowns occur^67^. Hence, an appropriate long-term perspective is imperative for the management of saline lakes.
4. Stakeholder engagement: The majority of saline lakes occur in dryland regions^68^, which are often located in the periphery, away from economic and political centres. Furthermore, the catchments of many saline lakes span across national boundaries. Such transboundary catchments combined with the high sensitivity of saline lakes to changes in their water balance can lead to intensified resource-use conflicts (e.g., between using water for irrigation in one country and letting it evaporate in the lake of a second country). Hence, active and broad stakeholder engagement, for example through transnational management boards that should include indigenous and other marginalised stakeholders, becomes a key requirement for the successful management of saline lakes.
5. Adaptive management and restoration: Current climatic changes have already affected many lakes^8,69^ and in most dryland systems, it will be impossible to compensate higher evaporation rates and reverse changes in lake levels. Hence, many saline lake systems will shift away from historical baseline conditions and show substantial changes in salinity and lake level dynamics, rendering comparisons with predefined reference states to be of limited use. Accordingly, management strategies and monitoring schemes need to be continuously adapted to account for changes in core ecosystem services and ecological communities.

Despite the complex challenges associated with the management of saline lakes, the notion of leaving them unmanaged is untenable. The recommendations provided here aim to address the raising environmental pressures and their detrimental impacts on saline lake^22,70^ by supporting the development of comprehensive monitoring and management frameworks.

### Forging a vision for future conservation of saline lakes

Although saline lakes belong to the most threatened ecosystem types worldwide, they are often marginalised in national and international conservation and policy agendas. Here, we demonstrate that saline lakes support a higher number of threatened species and a more diverse set of economically and culturally important ecosystem services than commonly perceived. These include often under-appreciated existence values and services such as the regulation of microclimates in drylands, the prevention of dust-storms or the hosting of highly diverse microbial communities. However, the value of saline lakes is also rooted in their important contribution to diversity at the genetic and ecosystem level. Securing the ecosystem services and diversity supported by saline lakes will require a proactive approach that addresses their unique challenges. Whilst saline lakes often exist in remote and arid regions, this geographical isolation may also present an opportunity for implementing innovative conservation strategies. By better representing saline lake ecosystems on national and international policy agendas, decision-makers can foster collaborative efforts to ensure their sustainable management. The resulting stakeholder engagement can be an effective strategy to reconcile diverging interests and forge a joint vision to safeguard saline lake and their valuable ecosystems.

## Methods

The requirements for including lakes in our study were long-term mean salinity levels above 1 g L^−1^ and that lakes were separated from the marine system by a physical structure that restricts species migration. We chose these criteria to be inclusive and cover the full bandwidth of non-freshwater lakes. Quantitative data collection was based on an international survey among saline lake researchers. The questionnaire of the survey comprised four sections covering ecosystem characteristics, conservation status and endangered species, ecosystem services and, threats to the lake (see Supplementary Data 5 – the template of the questionnaire). Most researchers working on saline lakes are aquatic ecologists, and we hence framed our questionnaire around ecosystem services. This concept has been established longer in aquatic research than the concept of nature’s contribution to people^71^, and we hence considered it as more robust basis for our data collection. Accordingly, we also simplified the categorisation of cultural ecosystem services to avoid introducing bias due to the diverse backgrounds of data-contributing researchers.

In total, the questionnaire contained 71 questions to attain impartial information (e.g., identity of threatened species) as well as expert-based evaluations (e.g., ranking severity of threats). We considered only complete surveys (*n* = 85) that provided answers to a core set of 37 compulsory questions. The remaining questions required specific quantitative information that varies largely in its availability across lakes and regions. Hence, these questions were considered as optional to avoid systematic biases in our database. Researchers tend to specialise on lakes with above average biodiversity and aesthetic value (reflected in the 43% RAMSAR sites in our data set), and their interest will naturally lead to scientific publications, partially explaining the high proportion of lakes with more than 20 publications in our data set (i.e., criteria for supporting research as cultural ecosystem service). Yet, this bias for more biodiverse lake should not affect the reported relationships between environmental characteristics and ecosystem services (Figs. 3, 4) and hence we are confident in the robustness of our results.

Threatened species were defined as species that were ranked on the IUCN Red List of Threatened Species as ‘Near Threatened’, ‘Vulnerable’, ‘Endangered’, ‘Critically Endangered’ or ‘Regional Extinct’ (assessed in January 2023, see Supplementary Data 4 for a list of species). We focused on threatened species because of their importance for conservation policy and because information on biodiversity across different taxa largely varied among lakes, and the use of more general biodiversity indices could have hence resulted in potential bias.

A phylogenetic tree was created using the NCBI Taxonomy ID of each threatened species^72^. The NCBI Taxonomy ID is a unique identifier assigned by NCBI for each species and includes taxonomic lineage information. Despite extensive species coverage, 16 of the threatened species highlighted earlier were not present in the NCBI database, and taxonomic lineage information could not be retrieved. In these cases, we identified a closely related sister species and used its unique NCBI taxonomy ID and sequence information present in the NCBI Taxonomy ID database. The tree was built using phyloT (https://phylot.biobyte.de/) using the default parameters (automatic determination of direction; cost matrix = 51% similarity; gap open penalty = 12; gap extension penalty = 3) for a global alignment with free end gaps. The tree was visualised using itol (https://itol.embl.de/).

### Expert consultation process

A second element of our data collection was a repeated expert consultation process organised amongst our co-author group of 67 saline lake researchers (34 male, 33 female) from 20 countries to identify (i) main characteristics of saline lakes that complicate their management, and (ii) synthesise best-practice recommendations for the design of management and monitoring frameworks. Expert consultations comprised of three steps.

First, experts were asked to provide their opinion on management and monitoring challenges in saline lakes in a virtual flipchart that facilitated expert communication (Appendix 1, Supplementary Fig. S1). The responses to these questions were then discussed as the second step in an online plenum meeting (32 participants) where participants identified three core questions for further discussion. These questions were: (i) Which characteristics make saline lakes so difficult to manage? (ii) What are the practical implications and challenges of these characteristics? (iii) What are the resulting general recommendations for the design of management and monitoring plans of saline lakes? These questions were then discussed in four parallel video-recorded breakout groups. A repeated design with multiple breakout groups addressing the same questions was chosen to elicit a wider range of complementary answers and reduce bias^73^. In the final step of the expert consultation, the transcription of the recorded answers (Supplementary Fig. S1) and a conceptual synthesis of the discussions were presented to all co-authors for additional amendments and to reach expert agreement.

### Statistical analyses

We evaluated the drivers of (i) the number of threatened species, and (ii) the number of different ecosystem services provided per lake in multiple regression analyses. Based on the sample size (*n* = 85), we selected nine potential predictors to be included in the regression analyses. These were lake surface area, volume, salinity (average of last ten years), variability of salinity (range of recorded salinity in last 50 years), water type (three categories: originating from seawater, soda-lakes which are alkaline with pH > 9.5 and inland non-soda lakes; Schagerl 2022), regular desiccation of the lake (binomial), formal protection of the lake (binomial) and remoteness (defined by the distance to the next city with > 100,000 inhabitants). Further, we included the interaction between volume and surface area to account for lake bathymetry (i.e., surface-volume ratio) effects, but did not consider other interaction effects to avoid overfitting.

In a full model, including all potential predictors, we checked for non-linearities and applied logarithmic or square transformation if necessary. Subsequently, we z-standardised all predictors to facilitate comparison of their effect sizes. The most parsimonious models were identified based on Akaike’s Information Criterion (AIC). Residuals were checked for normality, homogeneity of variance across ranges of response variables, and other remaining patterns. In the case of the regression predicting the number of threatened species per lake, we used a generalised linear model based on a Poisson distribution to improve residual diagnostics. Finally, we evaluated the impact of salinity on the provision of individual ecosystem services in a logistic regression. We included formal protection and the presence of endangered species as an indicator of high species biodiversity, which is frequently considered as supporting ecosystem services. Again, logistic, square, or unimodal transformation of the predictor were implemented if they substantially improved model fit (ΔAIC values of > 0.5). All analyses were implemented in R version 4.1.0^74^. R script is available as Supplementary Code 1. The UFZ ombudsman for good scientific conduct provided a confirmation that interview data collection, storage and presentation followed good scientific practices.

### Ethics & inclusion statement

Roles and responsibilities were agreed amongst collaborators ahead of the research, and the involvement of local researchers was discussed at the initial stage of the research. It was stated at the initial request that “Our goal is to collect information from approximately 50 lakes ranging in salinity from brackish to hypersaline conditions and located in different continents and climatic zones. The expected outcome of this survey is a paper co-produced by the current research community working on saline lakes. All researchers who provide data for at least one lake will be invited as co-authors to the resulting paper”.

### Reporting summary

Further information on research design is available in the Nature Portfolio Reporting Summary linked to this article.

## Supplementary information

Supplementary Information

Peer Review file

Description of Additional Supplementary Files

Supplementary Data 1

Supplementary Data 2

Supplementary Data 3

Supplementary Data 4

Supplementary Data 5

Supplementary Code 1

Reporting Summary

## Data Availability

The data gathered and used in this study are provided in the Supplementary Information/Data files (Supplementary Data 1–5).

## Code availability

The annotated R script is included as Supplementary Code 1.
