# Supplementary material for: The overlooked conservation values of saline lakes
Source: Nat Commun. 2026 Sep 3;17:9456. doi: 10.1038/s41467-026-76684-0 (PMC13542260; doi:10.1038/s41467-026-76684-0)
Supplement: Supplementary file 2 [file 41467_2026_76684_MOESM1_ESM.pdf]

Supplementary Information

The overlooked conservation values of saline lakes

Alfred Burian, Ramesh Wilson, Pavel Kratina, Ors Abram, Ekaterina Afonina, Javier Alcocer, María Belén Alfonso, Tania Anderson, Elena Anufriieva, Nurgül Balci, Luciana Gomes Barbosa, Bonnie K. Baxter, Balzhit Bazarova, Yuri Bazhenov, Michael Beckmann, Emil Boros, Svetlana Borzenko, Enrique H. Bucher, Antonio Camacho, Emilio O Casamayor, Xiaobo Chao, Peri Coleman, María Florencia Colla, Bindy Datson, Santiago Echaniz, Maria Eugenia Farias, Tadesse Fetahi, Carlos M García, Larisa Golovatyuk, Peter Hudson, Katia Hueso-Kortekaas, Jill Johnston, Bakhtiyor Karimov, Irina Kalioujnaia, Anastasia Komova, Lothar Krienitz, Seema Kulshreshtha, Ron Larson, Oksana Lipka, Evgeniya Matyugina, Quentin Mauvisseau, John Melack, Keely Mills, Daniel Morant, Edmundo Moreno, Zorigto Namsaraev, Aharon Oren, Christopher Rogers, David Ryves, Marta I. Sánchez, Alba Camacho-Santamans, Anna C. Santamans, Michael Schagerl, Elena Selivanova, Nickolai Shadrin, Somayeh Sima, Natalya Tashlykova, Katrin Teubner, Gazhit Tsybekmitova, Brian Timms, Svetlana Ulanova, Nicolás E. Vidal Quini, Alicia Vignatti, Wayne A. Wurtsbaugh, Charitos Zapitis, Thomas Zechmeister, Can Zhang, Egor Zadereev

Table of content

Supplementary Note 1: More specific discussion of management and monitoring standards in the EU
Additional tables and figures
References

Supplementary Note 1: More specific discussion of management and monitoring standards in the EU

In contrasts to freshwater systems, the level of management and monitoring standards for saline lakes largely diverge among EU member states. We provide here examples from two different countries (Austria and Spain) to provide critical reflections on potential improvements and strong points that can serve as model for other countries.

(i) Austria

Saline lakes in Austria are concentrated in the drier Eastern part of the country with a continental, temperate climate. A major step toward the sustainable management of these lakes is that Lake Neusiedl, the largest saline lakes is monitored under the Water Framework Directive of the EU (BGBl. II Nr. 99/2010) and a special monitoring program has been implemented for smaller saline pans. A further major achievement is that boundaries have been established that define a good ecological status of individual lakes and pans. Hence, the collected field data are used to evaluate the state of individual saline ecosystems in Austria.

Despite these accomplishments, several major challenges remain unaddressed. First, the monitoring of saline lakes fails to meet the advanced level that has been reached in the evaluation of freshwater systems. Monitoring of freshwater systems relies on both physio-chemical conditions as well as on biological status assessments. In saline lakes, however, monitoring includes primarily the evaluation of salinity and pH-values. Furthermore, target chlorophyll-*a* values have been defined. However, monitoring lacks the comprehensive evaluation of primary producers (macrophyte or phytoplankton) or invertebrates that is implemented for freshwater communities. Hence, the biological component of monitoring is comparatively poor in Austrian saline lakes, presumably also because there are no comprehensive regional bio-indicator indices that have been developed for this purpose.

Moreover, there are no saline-specific parameters included in current environmental monitoring frameworks (BGBl. II Nr. 99/2010). For example, the fundamental importance of monitoring water balances in saline lakes was highlighted[1]. Hence, indicators such as catchment-wide water balances or other hydrological indicators are central to evaluate the status of saline lake systems. Their inclusion in ecological and environmental status assessments of saline water bodies would substantially strengthen their power an efficacy to support management.

An additional challenge is that there are no actionable management interventions that are universally agreed upon and that can rapidly be implemented in case of emergency. This situation is exemplified by the case of Lake Neusiedl. The lake has experienced massive alterations of its catchment over the course of the last four centuries[2]. Currently, reductions in lake levels are a central issue of concern[3,4,5]. Proposed countermeasures include alterations in the management of an artificial channel that reduces maximum lake levels, redirection of new water sources to the lake or the artificial removal of sediments to increase the depth of the lake. However, these measures could induce strong trade-offs for the provisioning of different ecosystems services and could negatively affect either the conservation of the lake’s fauna and flora, the potential of agricultural production close to the lake or the regional tourism industry. A consequence of the resulting conflicting interests is that there are currently no clear actionable interventions that can be implemented in case of potential deterioration in the ecological status of the lake.

(ii) Spain

Spain is the European country with the highest number of saline ecosystems accounting for almost hundred inland saline lakes[6,7,8]. Most of them are temporary[9,10], with summer annual drying, but some are naturally or artificially[11] permanently flooded. Although their salinity fluctuates seasonally, some are typically mesosaline whereas others are permanently hypersaline[12]. These athalassohaline lakes are mainly endorheic, and are distributed within several lake-districts. These main lake districts are located in the regions of Andalucia and Aragon[13], as well as in Castilla - La Mancha[14] and Castilla y Leon. Magnesium sulphate is the main salt in mesosaline lakes, whereas this dominance turns to sodium chloride in hypersaline lakes[15,16], although sodium bicarbonate prevails in soda lakes. These lakes have a very characteristic flora[17] and specific relict fauna well-know from time ago[18], and hence the region exemplifies the diversity of saline lakes.

Because of their environmental relevance, saline lakes have been considered in the development of the European Water Framework Directive[19] by Spain. The classification of the lake type waterbodies in Spain uses system B, which includes environmental features such as conductivity, aridity index, hydroperiod, alkalinity, and others. These environmental parameters are used to distinguish among saline lakes and differentiate them from freshwaters systems. From a total of 30 lake types considered in the Spanish classification of "lake type" waterbodies, 4 correspond to inland saline lakes, two mesosaline (LT-20 permanent, and LT21 temporary) and two hypesaline (LT-22 permanent, and LT23 temporary). By considering them as waterbodies (official consideration as WFD targets for achieving their good ecological status), Type LT-20 includes 7 mesosaline permanent lakes, LT-21 26 mesosaline temporary lakes, LT-23 14 hypersaline temporary lakes and, finally, LT-22 includes the only permanent (relatively) deep (5.6 m) hypersaline lake in Western Europe, the Salada de Chiprana[20].

All these lakes have specific reference conditions and thresholds for the ecological quality ratio (EQR) for the same indicator variables used for the freshwater lakes in Spain: These include the same biological quality elements than freshwater lakes, namely phytoplankton, macrophytes (hydrophytes, helophytes and, further for saline lakes, halophytes) and benthic invertebrates, except fish, as the Spanish inland saline lakes are naturally fishless. Further, the assessment of their ecological status also includes the same physical, chemical (e.g. conductivity), and hydromorphological (e.g. hydroperiod length) variables than freshwater lakes. Thus, the requirements of achieving the good ecological status and its assessment system of Spanish saline lakes are equally stringent than any of the other lakes declared as waterbodies under the WFD. Consequently, it can be stated that in Spain, all saline lakes declared as lake waterbodies are, thus, equally considered as any other Spanish lake.

As including a complete set of variables in the assessment system, and thresholds related to the five quality classes of the WFD, the quality requirements of saline lakes are fully monitored and deviations from the good ecological status can be detected and, even more importantly, corrected with actions named within the river basin management plans. As such, these plans have designed and implemented actions to reduce trophic alterations, one of the main pressures suffered by inland saline lakes, mainly caused by the former inputs of sewage or, more recently, treated wastewaters[21]. Additional to an increased trophic status, these water inputs, together with agriculture run-off, cause drops of water salinity threatening the essential saline character of their waters, and determine changes in the natural hydroperiod that uncouple the natural biological cycles and the lake biogeochemistry. Moreover, abstraction from the aquifers have caused a drop in the natural groundwater feeding of some of these lakes, particularly in La Mancha, also changing lake´s natural hydrological patterns. So far, the river basin management plans, jointly with the nature conservation authorities, have designed a number of actions that permitted the improvement of the ecological status of an important number of Spanish inland saline lakes. Hence, the approach used by Spain could be considered as an example on how systematic monitoring and consolidated actions can help to safeguard ecosystem services and natural biodiversity supported by saline lakes.

Additional tables and figures

Table S1: Details of most parsimonious models that were established in regression modelling processes. Only models that yielded results that were better than null-models are listed. All predictors listed in formulars were z-scaled to aid interpretability of slopes.

Dependent	Predictors	Transformations	Formula	Model fit
Number of endangered species	x1 = salinity	x1 = squared	y = -0.14 x1 + 2.49	NA
Number of ecosystem services	x1 = surface area<br>x2 = remoteness	x1 = log	y = 0.99 x1 - 0.72 x2 + 4.77	0.39
Commercial use of lake water	x1 = salinity	x1 = u-shaped + logit	y = -0.36 x1 + 6.08	0.069
Use of lake for fishing	x1 = salinity	x1 = log + logit	y = -1.15 x1 + 1.92	0.353
Use of lake for salt extraction	x1 = salinity	x1 = log + logit	y = 0.70 x1 - 3.86	0.146
Presence of endangered species	x1 = salinity	x1 = log + logit	y = -0.45 x1 + 2.98	0.069

Table S2: Most important threats for saline lakes and most important impacts of climate change highlighted by saline lake researchers.

Most important threats	Count
Climate change	52
Land-use change	46
Natural resource use and exploitation, including:	42
- Water abstraction	27
- Overexploitation	6
- Mining	9
Pollution	26
Invasive species	6
Others	12

Most important impacts of climate change	Count
Increased evaporation	54
Reduced water inflow	35
Higher water temperature	35
Increased variability	25
Others	63

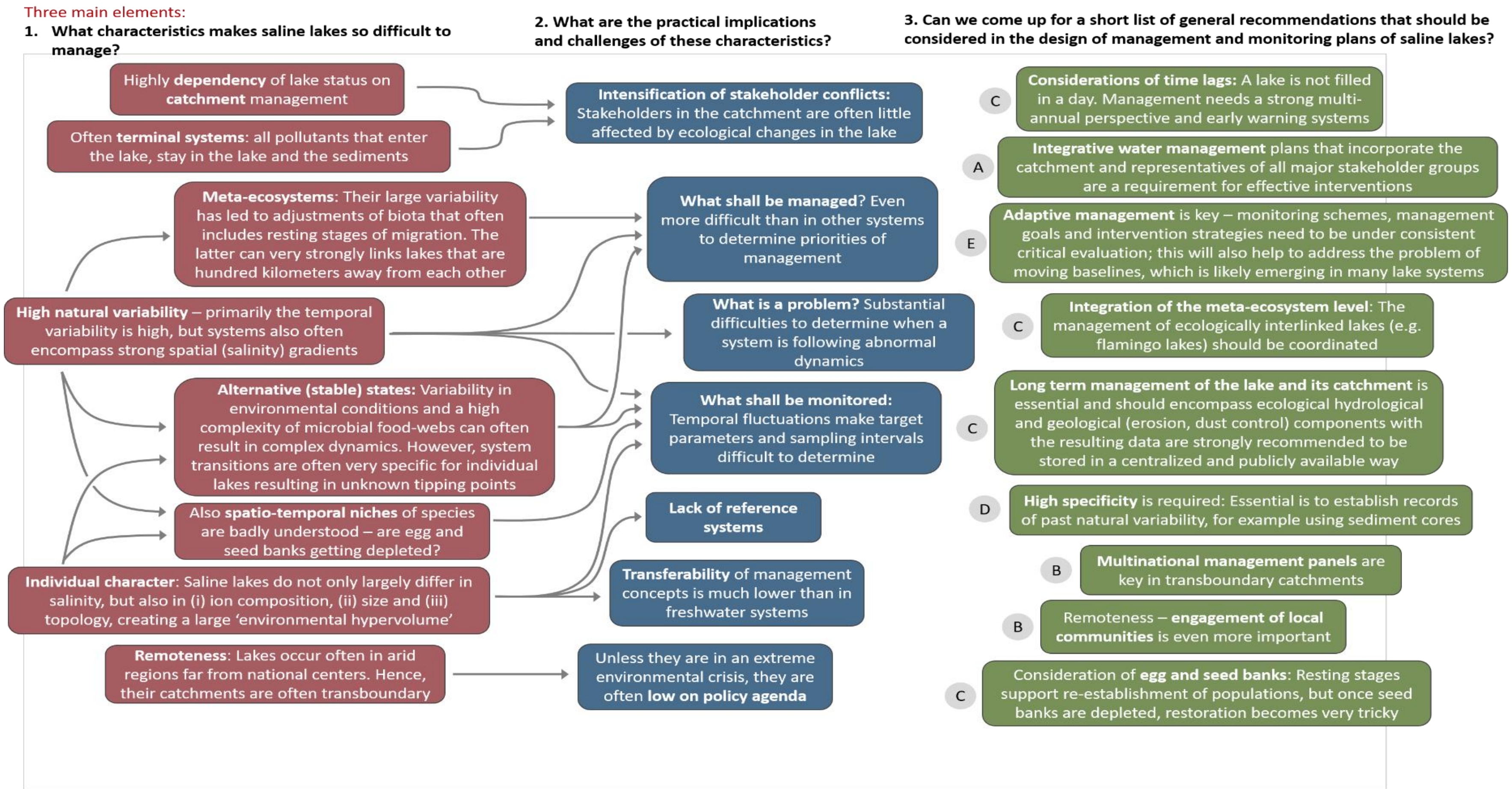

Fig. S1: Synthesis of the repeated expert group discussions on the three key questions (answers color red, blue and green, respectively) identified in the online plenum discussion. The answers to question 1 and 2 have been integrated into Fig. 5 in the main text. Answers to question 3 have been grouped into five core recommendations (Letters A-E). These five recommendations were (A) Integrated water management plans, (B) Stakeholder engagement, (C) Long term planning for meta-ecosystems, (D) Accounting for high specificity and (E) Adaptive management and restoration.

References

1. Wurtsbaugh, W.A., Miller, C., Null, S.E., DeRose, R.J., Wilcock, P., Hahnenberger, M., Howe, F. and Moore, J., 2017. Decline of the world's saline lakes. *Nature Geoscience*, *10*(11), pp.816-821.
2. Tolotti, M., Guella, G., Herzig, A., Rodeghiero, M., Rose, N.L., Soja, G., Zechmeister, T., Yang, H. and Teubner, K., 2021. Assessing the ecological vulnerability of the shallow steppe Lake Neusiedl (Austria-Hungary) to climate-driven hydrological changes using a palaeolimnological approach. *Journal of Great Lakes Research*, *47*(5), pp.1327-1344.
3. Cotera, R.V., Guillaumot, L., Sahu, R.K., Nam, C., Lierhammer, L. and Costa, M.M., 2023. An assessment of water management measures for climate change adaptation of agriculture in Seewinkel. *Science of the Total Environment*, *885*, p.163906.
4. Soja, G., Züger, J., Knoflacher, M., Kinner, P. and Soja, A.M., 2013. Climate impacts on water balance of a shallow steppe lake in Eastern Austria (Lake Neusiedl). *Journal of Hydrology*, *480*, pp.115-124.
5. Hackl, P. and Ledolter, J., 2023. A Statistical Analysis of the Water Levels at Lake Neusiedl. *Austrian Journal of Statistics*, *52*(1), pp.87-100.
6. Comin, F.A. and Alonso, M., 1988. Spanish salt lakes: their chemistry and biota. *Hydrobiologia*, *158*, pp.237-245.
7. Alcorlo, P., Baltanás, A. and Montes, C., 1996. Is it possible to predict the salinity of Iberian salt lakes from their conductivity?. *Hydrobiologia*, *330*, pp.137-142.
8. Martino, P., 1988. Limnología de las lagunas salinas españolas. PhD dissertation, Universidad Autónoma de Madrid, Madrid, Spain 264 pp.
9. Doña, C., Chang, N.B., Caselles, V., Sánchez, J.M., Pérez-Planells, L., Bisquert, M.D.M., García-Santos, V., Imen, S. and Camacho, A., 2016. Monitoring hydrological patterns of temporary lakes using remote sensing and machine learning models: Case study of la Mancha Húmeda Biosphere Reserve in central Spain. *Remote Sensing*, *8*(8), p.618.
10. Doña, C., Morant, D., Picazo, A., Rochera, C., Sánchez, J.M. and Camacho, A., 2021. Estimation of water coverage in permanent and temporary shallow lakes and wetlands by combining remote sensing techniques and genetic programming: application to the mediterranean basin of the Iberian Peninsula. *Remote Sensing*, *13*(4), p.652.
11. García-Ferrer, I., Camacho, A., Armengol, X., Miracle, M.R. and Vicente, E., 2003. Seasonal and spatial heterogeneity in the water chemistry of two sewage-affected saline shallow lakes from central Spain. *Hydrobiologia*, *506*, pp.101-110.
12. Hammer, U.T., 1986. *Saline lake ecosystems of the world* (Vol. 59). Springer Science & Business Media.
13. Guerrero, M.C. and De Wit, R., 1992. Microbial mats in the inland saline lakes of Spain. *Limnetica*, *8*, pp.197-204.
14. Ballesteros Navarro, B.J., Camuñas Palencia, C., Mejías Moreno, M., Camacho González, A., Sánchez Ramos, D., Albacete Carreño, L. and Rochera Cordellat, C.,

2018. *Lagunas mesetarias de La Mancha: Funcionamiento hidrológico, cultura y medio ambiente*. CSIC-Instituto Geológico y Minero de España (IGME).
15. De la Peña, J. A., 1987. Las lagunas de La Mancha: un ejemplo de sales en ambiente continental. In RACEFN (ed.), Bases Científicas Para la Protección de los Humedales en España. Real Academia de Ciencias, Madrid, Spain.
16. Camacho, A., Miracle, M.R. and Vicente, E., 2003. Which factors determine the abundance and distribution of picocyanobacteria in inland waters? A comparison among different types of lakes and ponds. *Archiv Fur Hydrobiologie*, *157*(3), pp.321-338.
17. Medina Domingo, L., 2002. Plantas acuáticas de las lagunas y humedales de Castilla-La Mancha.
18. Margalef, R., 1947. Estudios sobre la vida en las aguas continentals de la región manchega. Publicaciones Instituto de Biologia Aplicada 4: 5–51.
19. European Union (2000). Water Framework Directive (WFD) 2000/60/EC: Directive 2000/60/EC of the European Parliament and of the Council of 23 October 2000 establishing a framework for Community action in the field of water policy. Official Journal of the European Communities, L 327 (1): 1-72
20. Camacho, A. and de Wit, R., 2003. Effect of nitrogen and phosphorus additions on a benthic microbial mat from a hypersaline lake. *Aquatic microbial ecology*, *32*(3), pp.261-273.
21. Corrales-González, M., Rochera, C., Picazo, A. and Camacho, A., 2019. Effect of wastewater management on phosphorus content and sedimentary fractionation in Mediterranean saline lakes. *Science of the total environment*, *668*, pp.350-361.
