# Supplementary material for: The overlooked conservation values of saline lakes
Source: Nat Commun. 2026 Sep 3;17:9456. doi: 10.1038/s41467-026-76684-0 (PMC13542260; doi:10.1038/s41467-026-76684-0)
Supplement: Supplementary file 3 [file 41467_2026_76684_MOESM2_ESM.pdf]

nature portfolio

Peer Review File

The overlooked conservation values of saline lakes

Corresponding Author: Dr Alfred Burian

This file contains all reviewer reports in order by version, followed by all author rebuttals in order by version.

Version 0:

Reviewer comments:

Reviewer #1

(Remarks to the Author)
Comments on the manuscript
The overlooked conservation value and ecosystem services of saline lakes

The most noteworthy results are the compilation of many data from 84 lakes in the wide category of saline lakes, and the effort by a large number of experts worldwide to discuss about the challenge of finding common guidelines to management and conservation.

On the other hand, this work analyses systems that are neither marine nor freshwater, the two major reservoirs of diversity and services that have been studied the most. The ms. analyses systems across the broad gradient of saline lakes, that have received much less attention in terms of resource management, formal protection and proposal of a set of rules of use in view of their potential exploitation.

The manuscript is properly written. However, and probably due to the effort to widen the analysis, the heterogenous origin of data and opinions and the complexity of the range of existing aquatic ecosystems, the final organization of the main text results somehow confusing with many ideas mentioned in a scattered manner that, however, are placed in an orderly scheme in the figure 5. These ideas could have been better organised in the text adding some clear guidelines to future management.

Overall, the manuscript seems publishable to me, although I think there is room for improvement, especially in terms of initial framework, writing and organization. So, my opinion is that it should be reviewed and improved before publication.

I will write a relatively long reflection on some points that would be worth considering, with the request that they be used whenever necessary to improve the message of the manuscript. After that, I will open another section ordered by line number in the draft with some more specific instructions on smaller details. Please do not interpret length as a synonym for greater criticism, but rather an effort to inspire some changes in order to improve an article whose content is of interest to the conservation of this type of systems.

Reflections

General

The work focuses in "saline lakes" and follows one of possible criteria to include lakes in the analysis, although some other introduction or selection could have been made (explained below). Abstract itself emphasizes as main objective and message that policy agendas do not include the protection and management of saline lakes because it is thought that they do not provide many ecosystem services and that they have little diversity. This seems to force them to present services (as fisheries etc) and high values of (known) diversity as key elements of the main arguments to protect them. So, it draws attention to the importance of conserving saline lakes based on these two pillars that end up being somewhat simplified: diversity based on the number of species on red lists, mainly vertebrates, and a list of services that, at times, only reflect a current reality but do not provide clear clues about the true potential of these systems and the caveats when managing services. The good thing is that they analyse reality through existing data. Nevertheless, since a group of world experts who are well aware of the potential of these systems has been brought together, there is a lack of clear and highlighted text speaking of what is not seen, precisely because these systems are not well known and (probably) are not being used as

they could be. The analysis of existing data is well fulfilled in results, but these other ideas could have been more clearly added in a specific and well-organised section (now are scattered) as part of discussion and proposal of future management.

Some ideas to consider

1-Introduction and unified framework approach for aquatic systems

If we accept the world's aquatic ecosystems as a unit in which we can identify ecotones or boundaries separating more or less clearly differentiated communities/systems, two large poles can be identified that support the greatest diversity of aquatic organisms on the planet: the freshwater and marine habitats. There is an evolutionary and salinity difference, but they share homeostasis and stability in the physical environment as a characteristic, as well as a tendency for the community structure to be determined by biological control. On the other hand, there are both thalassic or marine environments and athalassic or continental ones that also have evolutionary and salinity differences, but they share a common feature: they are not physically stable. The fluctuations and unpredictability of environmental variables lead to the selection of communities where only the most tolerant organisms, adapted to frequent changes, can thrive. So, in my opinion, the target systems could have been not exactly "saline" systems (that include the sea), but astatic systems. In fact, the ms includes three categories of lakes, coastal lagoons, soda lakes and other inland saline lakes (that would include many other). But it would have been perfectly plausible to consider ephemeral or temporary freshwater systems in which, despite maintaining a salinity that changes little around fresh and subsaline, there is no high diversity and there is a set of species very different from those of permanent lakes due to tolerance to desiccation and short hydroperiod (e.g. many branchiopods and amphibians). Many saline lakes, except some permanent, are also temporary and almost all are fluctuating systems. This leads to a wide set of communities and sets of environmental variables, that complete the whole n-dimensional space of factors that the ms depicts in the upper right corner of figure 5.

Choosing factors or variables that define the limits of these physically controlled systems is nothing but an arbitrary decision. You can choose one of the axes that you consider very relevant, such as salinity, but whichever one you choose, it will be an arbitrary decision that can be justified based on where we think the main "ecotones" are established. Salinity is one of the axes, but fluctuations, degree of permanence (hydroperiod) and some others are possible. Many may be possible and it is a matter of debate to decide which is most appropriate. Let us follow the path of salinity. But, even when choosing salinity, some lines about this unified framework approach for aquatics systems, could be added to the introduction/abstract (i.e. the key of deviation of the traditional freshwater and marine systems that, in the case of marine, is not salinity).

Similarly, the formal expression that the n-dimensional space formed by these axes of variation (salinity, degree of eutrophication, size, hydroperiod, etc.) is the central element of study rather than certain individual lakes or lake types. This would better reflect the reality that the systems are rather large regions in space, interconnected and heterogeneous and submitted to wide time scales changes (daily, seasonal and interannual) which provide identity to each system, not certain values of physical variables and a fixed list of species. This is more or less already expressed in the text but is not clearly the central element. The main idea to highlight is that all the diversity accumulated over a wide range of time and space in a connected region should be added together to give a picture closer to reality. This is important, for these more realistic values of diversity will increase vs alpha-diversity values measurements in just one of the states.

2- Selection of systems and criteria.

Once the axis "salinity" has been chosen, the selection of the lakes finally analysed is relevant to the result, so we can discuss it somewhat. In principle it has been carried out by taking the value of 1 g L-1 as the lower threshold, but discarding a large part of the (saline) marine systems (coastal and oceanic waters) although some coastal lagoons are included. So, the criterion "salinity" is mixed as another consideration (i.e. do not include seawater) is overlapping. This criterion is still acceptable but, like other possible ones, it is arbitrary and other decisions could have been made. These could have led to another view of the results.

For example:
- Discarding all the communities of thalassic/marine origin (since the typical communities and species are different from the continental ones even with the same salinity values due to evolutive reasons). (I do not see it as necessary, but it would have been a perfectly plausible decision)

- The paradigmatic meeting between the two mentioned types of homeostatic waters occurs in transitional waters on the coasts, estuarine waters, which due to this boundary characteristic and the dynamics of hydrodynamics become waters with a salinity deviated from both large homeostatic poles and are also fluctuating and variable. In this estuarine framework, it is known that works such as Remane & Schlieper (1971) identify an "ecotone" around waters of relatively low salinity (3 to 10) that host a minimum of diversity: a divergent boundary between freshwater and marine systems. Looking for a criterion based on observations similar to this, in which an "ecotone" is identified, the compilation could have moved its limits to e.g. 3-5 g L-1. This decision would have led to considering hyposaline lakes as the beginning of the salinity gradient studied rather than also including subsaline ones (<3).

These two options (which do not necessarily have to be taken if justified) would have consequences on the results presented, such as, for example, the detection of a considerably lower diversity and a relative loss of importance of some services, such as fisheries. So, results and conclusions depend on these decisions on limits, leading to the need to justify or relativize the conclusions under discussion.

The reality is that it is difficult and arbitrary to draw boundaries between categories of aquatic systems because, although there are sometimes very marked ecotones, they are actually open communities.

In the current version, lakes > 1 g L-1 have been included in the analysis. However, in table S4.1, which describes the characteristics of the lakes to be studied, there is one with only 0.17 g/L (fresh water, L. Epecuen) that should be eliminated of the analysis (or review the value of salinity) and there are 14 with nominal salinity ≤4 with salinity ranges that take the waters to limits close to the lower border of subsaline waters. Figure 3 shows graphically how the “fisheries” service is clearly restricted to this type of waters bordering on the freshwater area and this selection will have also consequences on diversity. So, either reduce the list or mention this in discussion of results.

4- Value of ecosystems based on diversity

Do we really have to evaluate systems only by the numerical diversity of already known species? by the number of taxa on a red list without any additional consideration?

I know that these are the tools we have to raise well-founded numerical answers, but perhaps in the discussion we should highlight other values beyond that. The reader should see that the analysis is acceptable, but also based on conventional criteria and, at least, open to criticism.

First of all, although recognised in the text, and especially in high salinity lakes, aquatic invertebrates and unicellular organisms are crucial explaining biodiversity. But they are almost not addressed in the study (although are widely known by the authors). Besides that, species still to be described by Science are probably in a larger percentage than in marine or freshwater systems due to less research effort. These facts (especially first) could add a lot value to diversity beyond that shown in figure 2 and should be especially highlighted even in the figure caption for hurried readers. This would add to the comment already made about seeing diversity as the sum of a whole series of states.

Also, it is well known that the increase or deviation in salinity values (above that of freshwater or the sea) leads to a significant decrease in diversity. Should we say that the broad spectrum of saline lakes has a lesser value?

Perhaps it should be mentioned that equating the value of a system to the known species diversity could be a simplification. This can be the case of hypersaline lakes, as well as other extreme systems on our planet: Extreme environments on the Earth tend to be very little diverse, but they host unique organisms, capable of carrying out processes that are not redundant within the framework of the biosphere and, hence, are of high interest. So, a limited idea of species diversity (as represented in figure 2) is not a complete measure of the value of these ecosystems. Much of these systems have behaved as competitive refuges for highly-tolerant species, guarding very unique and valuable genes in the context of physiological adaptations. Let us not forget that one of the reasons for preserving biodiversity is to maintain a greater degree of resilience of the biosphere in the event of changes. The genes of microorganisms and, sometimes, very halotolerant metazoans (e.g. Artemia spp) guarantee the planet's capacity to adapt to changes. A first-order ecosystem service could be that of constituting gene reservoirs capable of producing and growing in very severe environments.

Another note on diversity would be to consider the different levels of it, especially beta-diversity or habitat diversity. Coming back to the consideration of the multidimensional space that define these systems, a goal of conservation management could be to preserve not only some alpha-diversity linked to a specific coordinate or to a specific space and time, but rather, guaranteeing regional conservation of beta-diversity.

5-Ecosystem services

According to the summary, there is another pillar supporting the arguments for paying attention to the conservation of the treated systems: the services they provide to society. The identification of ecosystem “services” as a very relevant requirement in the society in which we live, may urge the inclusion of fisheries (and other included in figure 3 and table 1) as something fundamental, but, in my opinion, we must dare to claim the value of the saltiest lakes without fear that the potential services are not so evident.

As Figure 3 shows with colour coding, services can be classified into categories (provisioning, supporting and regulating and cultural). But there is also a prior distinction among them. Pollution is identified as a stressor in the text, and at the same time “wastewater discharges” are considered an ecosystem service and added to a list of values and advantages that lead to its conservation. Although it can be a fact, I think the text should lead the reader to think that the difficult balance between pressures/impacts/stressors and ecosystem services and their regulation is a key for management. Let’s say that they are convenient because they are actually being used for the identified services but some sentence should always remember that the basis of their conservation is to intelligently manage those services, since some are, at the same time, clear impacts. However, other services do not cause as much impact or are even provided by the systems simply functioning naturally (e.g. dust retention, carbon sequestration and many cultural and scientific ones). Exposing some of these services only as an advantage or value but not as a possible source of impact can be confusing if not worded appropriately. So, many of the ecosystem services must be clearly regulated in the management process so as not to become a threat, pressure or stressor, in short, to be sustainable.

Table 1 and discussion on it. Although it is clear that this is a compilation of current quantified services (e.g. t year-1 km-2…)

there are no uses of extraction of products related to non-fish biomass, whether invertebrates or products such as -carotene or glycerol that can be obtained, as well as a mention in the text of the future potential related to microbial processes, this in part appears in figure 3. The search for innovative new services and this more diffuse service linked to conservation of valuable genes can also be mentioned beyond the current quantified and more widespread ones.

These reflections could lead to relatively brief changes in actual redaction but I think that could be necessary in the evaluation of the importance of these lakes.

Some comments on smaller details (in order of lines)

Line 113. Change to Sustainable

Line 117. Abstract. See general discussion. Just a suggestion to add some comment not only on "red-listed" diversity but also the consideration that many taxa are not considered in this analysis as information comes mainly from vertebrates, as well as a brief mention to the uniqueness of the gene pool in the biosphere context especially in the saltiest lakes.

Line 135 (and table S4.1) review either the limits of the analysis (>1 gL-1), the real value of nominal salinity (0,17 g L-1) of Lake Epecuen (S. America) or eliminate it of the data pool (also species and services etc).

Line 138 (and figure 5) Just a suggestion to add the axis of hydroperiod (degree of permanence or frequency of desiccation) to the small graph of multidimensional space top right. It is an ecological sound factor determining the type of aquatic community (tolerance to desiccation) and giving uniqueness to the lakes and list of species, even more than bathymetry or other factors already depicted.

Lines 158-160. As already said, when aquatic systems are considered, there are two major sources of diversity and ecosystem services: continental freshwater systems and marine systems (the latest are never mentioned and win in the salt extraction and fish harvest categories). It is difficult to argue that salt lakes provide a comparable level of services or species diversity in a somehow competitive comparison. This is why I believe that the uniqueness of the species and the services they currently or potentially provide should be highlighted, as well as the innovative potential of new services.

Lines 165-170. Use these argumentations in discussion of results when both the diversity and the current services are valued.

Line 190 and 201-205. As the assessment of biodiversity in the broadest sense in these systems is a fundamental theme underlying this article, it is clear that an analysis of red lists full of vertebrates, some of which do not have a critical dependence on water characteristics comparable to aquatic organisms, is insufficient. This fact is acknowledged in this section, but perhaps it should add a recommendation of good practices for managers to assess the distribution of invertebrates. In fact, the addition of aquatic invertebrates to a red list or ascribing them to an IUCN threat category is not comparable to that followed for many vertebrates, but should rather be based on the area of occupancy of the species, i. e. to analyze the IUCN criterion B2, see for example García de Lomas et al (2007 -DOI 10.1007/s10750-017-3322-0). All of this without forgetting to identify unicellular organisms with a future potential to provide new ecosystem services.

Line 196. (cited Figures) Figure 2 appears in the pdf as pixelated with low resolution (e.g. species names). Try to put one with a higher resolution that when enlarged on the screen allows you to appreciate more detail.

Line 211. The tendency for the diversity to decrease with salinity is well known but, at the same time, as already mentioned, organisms' specialization increases. Physically controlled systems, considered as competitive refuges, but also reservoirs of unique functions, are naturally not highly diverse, but when considering what needs to be conserved, the number of species should not be considered alone.

Line 219. And the same goes for the size of lakes (or other factors in the n-dimensional space defining types of aquatic systems). It is true that large lakes or permanent lakes can reach a greater number of species, but biodiversity must be analysed in all its aspects. In this case, the level of beta-diversity or habitat diversity is crucial. We should consider the set of aquatic systems as a whole. Considering the global set of species along all the gradients in that multidimensional system and the balance of all those systems (the search for the preservation of beta-diversity) is a desirable goal. The same applies to consider the sum of species throughout a year or series of years in a set of interconnected lakes rather than diversity measured in a punctual state.

If we are guided only by the numerical value of their alpha-diversity to prioritize ecosystems conservation, we may be simplifying things and opening a door to lose valuable sets of species. In the current redaction of the ms. the importance of just "diversity" mainly in the sense of number of species in a local site or sample (alpha-div) and the services can lead to forget other valuable elements justifying conservation. It is therefore advisable to convey that what is desirable is the conservation of the entire set of states in the space of environmental gradients, all the resulting communities and the entire range of sustainable and conservation-compatible services, whether current or potential.

Lines 235 and 244. Pollution is identified as a stressor and at the same time "wastewater discharges" are considered an ecosystem service and added to a list of values and advantages that lead to its conservation. OK. That's a fact. But I think the text should lead the reader to think that the difficult balance between pressures/impacts/stressors and ecosystem

services and their regulation is the key. Some sentence should remember that the basis of their conservation is to intelligently manage those services, since some are, at the same time, possible impacts.

Line 239 Review “indivudal”

Line 260. arctic instead of artic

Line 292 Review “regulationg”

Line 305 Review “salin”

Lines 325-327. This fact (complexity of states not only from lake to lake but also within a single lake, see e.g. Garcia et al 1997 doi.org/10.1023/A:1002983723725) is identified as a “difficulty” in managing saline lakes (perhaps ecosystems at a global level). But it is a fundamental reality: it is not a species, nor a lake, nor a single state that society must preserve but these dynamic and heterogeneous processes or functioning. The challenge for the future is to design management schemes and procedures that take this feature into account in an integrative way.

Line 338 Review “buissnes” and “polical”

Line 410 and others (Methods)
A good effort to gather data from many lakes around the world and to gather the opinions of various experts through targeted questionnaires. At the same time, it is recognized that it is very complex to organize so much and such heterogeneous information. The text of the entire manuscript sometimes loses order and organization perhaps due to this, but in general it is good and I see no objections.

Figures and tables

Already mentioned: Figures could be designed to see details better. This is especially necessary for figure 2 that should be included in a much higher resolution. Currently much people try to enlarge figures in a digital platform (tablet, computer…) and this action in the actual pdf copy lead to blurred pixels.

Figure 3 (comment): I do not understand well “Formal Protection” as a Service… perhaps this could be explained in the text. The fact that a lake or basin is protected by regulations does not make sense to me whether it is a service that the ecosystem provides to society or vice versa. But I am not used to thinking in services and do not know recent trends defining them. At the same time, it is curious to observe that a set of regulations about the limits of the complete set of services is a service itself.

Figure 5 (comment): This figure is the core of conclusions and I like it. It summarises well many things that appear somewhat more scattered in the text. It is much more satisfactory outlining the challenges of management in that n-dimensional space, a characteristic that should be highlighted. But, few clues as to how to solve these challenges, I think that some trends or proposals should be written in the text as already pointed out. Perhaps educating the manager in integration and the existence of this complex set of states and factors and the need to see sets in space and time based on long-term monitoring directed to connected systems (which in the graph have been identified as Meta-ecosystems).

CMG

Reviewer #2

(Remarks to the Author)
Review of NCOMMS-25-03940

The manuscript of Burian et al. is based on the premise that saline lakes are more important for providing biodiversity and ecosystem services than previously acknowledged. While I agree that saline lakes are understudied aquatic ecosystems that deserve more investigation and attention of the scientific community, I find most of the study’s results overstated and sometimes even misleading.

One example is already in the 1st sentence of the manuscript. There, the authors claim that “saline lakes are a diverse group of ecosystems that account for over 45% of the global inland water volume”, citing the publication of Messager et al. (2016). Thereby, Burian et al. try to emphasize the global quantitative importance of saline lakes. Upon a closer look, figure 1 the paper of Messager et al. reports 44% of global lakes to be “large salt lakes” and I assume the number cited above refers to this figure. However, 41 of those 44% are represented by the Caspian Sea alone. As a consequence, only 3% of global lakes are salt lakes other than the admittedly huge, but single water body of the Caspian Sea.

Without doubt, saline lakes are extreme environments. Such extreme environments typically have low biodiversity in terms of metazoans, although the authors correctly state that microbial diversity in saline systems can be high. First of all, the low biodiversity of metazoan life in extreme environments is neither a novel finding nor surprising, considering that only few specialized plant and animal taxa can survive under the extreme osmotic stress of saline and hypersaline lakes. If plant and animal diversity is low and saline lakes are admittedly under strong pressure from direct anthropogenic and indirect (climate-change related) stressors, the effect is that a high proportion of the population status of the metazoans in those vulnerable

systems is critical, leading to the inclusion of those taxa in the red lists of endangered taxa. However, the study's line of arguments becomes self-contradictory here. As noted above, the authors emphasize the saline lakes' high microbial diversity, thus making saline lakes highly diverse ecosystems. But microbes are not covered by red lists of threatened species. Nevertheless, the authors argue that the high proportion of threatened (red list) animal species found in saline lakes is evidence for the – as they argue overlooked - high biodiversity of saline lakes. As such, this line of reasoning does not make sense and the study states in line 193 that the saline lakes in their study hosted a median number of only three threatened species. Although I do not dispute the value of conserving saline inland waters, these low numbers cannot be considered proof for the claim that saline lakes harbour a large number of threatened aquatic biota, as made by the manuscript.

Another key claim of the paper is that saline lakes provide particularly relevant (cultural and economic) ecosystem services. Looking at the list of investigated ecosystem services in this study however, all of them (with the exception of salt mining) are ecosystems services that are provided by all kinds of lakes and not specific to saline lakes at all. While I will not dispute that conserving the world's lakes and their ecosystem services (which includes saline lakes) is of utmost importance, I do not see why the saline lakes should deserve this particular emphasis over lakes in general. I would even presume that non-saline lakes could potentially provide even more ecosystem services than saline ones, in particular services related to drinking water and fisheries (as fish densities are much higher in non-saline lakes, as stated in line 264).

Last but not least, not a single of the proposed "best-practice recommendations for sustainable management of saline lakes" (line 128) is exclusive to saline lake systems. Most of them – while not incorrect – are very generic and probably applicable to inland waters in general.

Version 1:

Reviewer comments:

Reviewer #1

(Remarks to the Author)
Remarks to authors
After reading the revised manuscript, I have noticed that my review work has been appreciated by the authors (for which I am sincerely grateful, as preparing those recommendations took quite some time and was based on personal ideas developed over years—something rarely acknowledged). Most of the ideas I had previously noted as missing from the text have now been incorporated into specific lines or paragraphs. From this perspective, and also appreciating the new organisation of the section on examples of good practices and the changes made to Figure 2, to mention just one example, the manuscript has improved. However, in my opinion, these aspects could have been developed a little further and, above all, the central conceptual framework or main message of the potential article has not changed substantially.
The manuscript continues to insist on a message that assumes that the conservation of systems is justified by their diversity values (quantified solely through larger or vertebrate species, with particular attention to red lists) and by the ecosystem services they provide. As I mentioned in my first review, there are two large groups of ecosystems that are better studied, more stable, and at the same time much more diverse, that are used more intensively by humankind globally. In fact, they are vital for oxygen supply, gas exchange and other biological and physicochemical processes—namely, freshwater and marine environments—so the central argument loses strength as a key justification.
It is a fact that these other two types of environments exhibit greater diversity, even if, as is now discussed (and was less so in the first version), global biodiversity is considered taking into account genes, habitat-level or beta-diversity, and a more integrated vision across longer time scales, not just on point inventories.
Regarding ecosystem services, it is clear that fishing (one of the first services that comes to mind for the common responsible for benefits when aquatic systems are mentioned) is here concentrated in lakes in the hyposaline and subsaline margins, rather than across the broader salinity gradient of continental saline lakes. Other services expressed in Figure 3 and later in Table 1, such as wastewater discharge, tend to be provided more massively and are more easily managed by seas and large lakes, without posing the intolerable pressure or impact found in many saline lakes of smaller volume, which would need careful analysis and management. Research, recreational value, and the presence of species of interest are not a particular strong point for this type of systems either, since many of these services in freshwater and marine systems represent higher and mostly far superior values. Even in the case of salt extraction, marine salt pans are of much greater importance. Therefore, the very conception of the article (namely, presenting these two pillars -diversity and services- as solid arguments for conservation) still does not seem to me the most satisfactory approach to demonstrate their value, at least not the value they deserve.
I believe I made it clear in my first review that I am fully convinced of the need to conserve these systems and that I consider them extremely valuable. It is true that the idea I mentioned about the uniqueness of the genes of highly tolerant organisms, which contribute resilience to our biosphere, has been incorporated into a paragraph in the new version, which I greatly welcome. However, assuming as the foundation of everything, that ecosystems are valuable only, or just to the extent that they provide numerous quantifiable services to humankind or host a considerable number of species already included on red lists, seems to me, personally, a kind of concession to a modern social consensus and prevailing evaluation frameworks that, sometimes, are susceptible to some criticism. It might be worthwhile to add a brief critical note on this widespread perspective, even daring to touch upon a somewhat romantic notion related to their intrinsic values by using, for instance, the uniqueness of those genes and processes. Personally, I always find it somewhat strange to have to justify the conservation of ecosystems and species based on their participation in "beneficial" processes, and I sometimes feel a little outside the

established system. In that sense, another possible approach would be to present the fraction of diversity related to vertebrates, that is measurable and easy to quantify, and the services they may provide as an added value rather than as the main argument. This could even be reflected in the title (if it were up to me, I would leave it as "The Underestimated Conservation Value of Saline Lakes"). But now the title in fact, makes services the central foundation of the manuscript.
In any case, as I have hinted, I feel that, after a long time studying aquatic systems, I have observed changes in how society as a whole perceives the value of ecosystems, and at times I have not been able to adapt fully to some of these changes. Thus, there is a need for the values to be easily quantifiable and readily integrated into economic or social models by a sector of society that does not perceive the "invisible", as already briefly mentioned in the new version. I am fully aware that this viewpoint of just a person may be legitimately criticised by the authors from the 58 prestigious institutions that have signed the manuscript. I also know it may or may not be shared by the editors. Therefore, I do not consider myself an infallible judge. For that reason, I will not issue a definitive verdict indicating whether I accept or reject the manuscript. However, my personal opinion is that it can at least be improved (it remains open to revision), and I believe its focus and structure could be reconsidered to the point of turning it, from my perspective, into an almost entirely new article. So I will qualify it as "need major revision" again.

Carlos M García
University of Cádiz
Spain

Reviewer #2

(Remarks to the Author)

I appreciate the authors' detailed response to the comments I had raised on an earlier version of their manuscript. They have clearly amended and improved the style of their manuscript. I do not find anything technically wrong with their analysis. Still, the sole purpose of this manuscript is to raise awareness on the understudied saline inland waters. While this is a valid opinion, there is not much scientific novelty in this paper that would provide significant advancement to the field.

Reviewer #3

(Remarks to the Author)
I have read this ms and, at the editor's request, I focused on the expert judgment aspects. The paper is well written. Expert judgment in this case is not forecasting values or quantifying uncertainty; it is a simple preference survey. The experts are identified as co-authors. I see no red flags or causes for concern here.

Two minor editorial remarks: (1) in the abstract there ae 84 saline lakes, but line 227 mentions 85 saline lakes. (2) in line 292 "a" should probably be "are".

Version 2:

Reviewer comments:

Reviewer #1

(Remarks to the Author)
About the general value of this manuscript I wrote in former reviews. Now, having read the new revised manuscript, I appreciate the inclusion of new paragraphs that encourage the reader to reflection and highlight valuable aspects of saline lakes that are not always easy to quantify at present. In my opinion, these aspects should be mentioned facing to future research that takes them into account. Overall, I have noticed a substantial improvement in the writing, which, combined with the extensive quantitative data already presented, leads me to think that the manuscript can be published in its current form. I am very grateful to the authors for considering the ideas I shared with them, including the slight change to the title.

I therefore have no objection to its publication.

Open Access This Peer Review File is licensed under a Creative Commons Attribution 4.0 International License, which permits use, sharing, adaptation, distribution and reproduction in any medium or format, as long as you give appropriate credit to the original author(s) and the source, provide a link to the Creative Commons license, and indicate if changes were made.
In cases where reviewers are anonymous, credit should be given to 'Anonymous Referee' and the source.
The images or other third party material in this Peer Review File are included in the article's Creative Commons license, unless indicated otherwise in a credit line to the material. If material is not included in the article's Creative Commons license and your intended use is not permitted by statutory regulation or exceeds the permitted use, you will need to obtain permission directly from the copyright holder.
To view a copy of this license, visit https://creativecommons.org/licenses/by/4.0/

Point-by-point response to reviewers' comment:

Reviewer #1 (Remarks to the Author):

Comments on the manuscript: The overlooked conservation value and ecosystem services of saline lakes

The most noteworthy results are the compilation of many data from 84 lakes in the wide category of saline lakes, and the effort by a large number of experts worldwide to discuss about the challenge of finding common guidelines to management and conservation. On the other hand, this work analyses systems that are neither marine nor freshwater, the two major reservoirs of diversity and services that have been studied the most. The ms. analyses systems across the broad gradient of saline lakes, that have received much less attention in terms of resource management, formal protection and proposal of a set of rules of use in view of their potential exploitation.

The manuscript is properly written. However, and probably due to the effort to widen the analysis, the heterogenous origin of data and opinions and the complexity of the range of existing aquatic ecosystems, the final organization of the main text results somehow confusing with many ideas mentioned in a scattered manner that, however, are placed in an orderly scheme in the figure 5. These ideas could have been better organised in the text adding some clear guidelines to future management.

Answer: Thank you for the thorough and extensive review of our manuscript. We very much appreciate the invested time and the constructive criticism of our work. Your comments have motivated us to implement several major improvements:

(i) We now clearly highlight the high diversity of saline lakes at the ecosystem (see Line 213-228). We strengthened our argumentation by referring to the CBD, where the ecosystem level is highlighted as one of three key components of biological diversity and by implementing a new analysis that highlights the high beta-diversity of saline lakes (Fig. 2)

(ii) We followed your advice to account for ecosystem services that are difficult to quantify. We now address them in a new section (see line 289 – *the truly hidden services of saline lakes*)

(iii) We followed your recommendation and the recommendation of reviewer 2 and reworked the best-practice recommendations (line 325 to 401). Here, we modified e.g. Fig. 5, following your suggestions and changed the text to highlight why our recommendations are specially relevant and pressing for saline lakes.

We are very confident that together with additional adjustments to address minor comments, this has helped us substantially to improve the quality of our submission.

Overall, the manuscript seems publishable to me, although I think there is room for improvement, especially in terms of initial framework, writing and organization. So, my opinion is that it should be reviewed and improved before publication.

Answer: We are glad that our work found your principal appreciation.

I will write a relatively long reflection on some points that would be worth considering, with the request that they be used whenever necessary to improve the message of the manuscript. After that, I will open another section ordered by line number in the draft with some more specific instructions on smaller

details. Please do not interpret length as a synonym for greater criticism, but rather an effort to inspire some changes in order to improve an article whose content is of interest to the conservation of this type of systems.

Answer: Again, thank you for your efforts! We very much appreciated the detailed feedback!

Reflections

General

The work focuses in "saline lakes" and follows one of possible criteria to include lakes in the analysis, although some other introduction or selection could have been made (explained below). Abstract itself emphasizes as main objective and message that policy agendas do not include the protection and management of saline lakes because it is thought that they do not provide many ecosystem services and that they have little diversity. This seems to force them to present services (as fisheries etc) and high values of (known) diversity as key elements of the main arguments to protect them. So, it draws attention to the importance of conserving saline lakes based on these two pillars that end up being somewhat simplified: diversity based on the number of species on red lists, mainly vertebrates, and a list of services that, at times, only reflect a current reality but do not provide clear clues about the true potential of these systems and the caveats when managing services. The good thing is that they analyse reality through existing data. Nevertheless, since a group of world experts who are well aware of the potential of these systems has been brought together, there is a lack of clear and highlighted text speaking of what is not seen, precisely because these systems are not well known and (probably) are not being used as they could be. The analysis of existing data is well fulfilled in results, but these other ideas could have been more clearly added in a specific and well-organised section (now are scattered) as part of discussion and proposal of future management.

Answer: We now highlight 'unseen' and potential future ecosystem services that we cannot quantify, but that are important in a dedicated section of the manuscript. Specifically, we highlight (i) the value of existence values e.g. resulting from microclimate regulation, (ii) their role as maintaining a gene pool adapted to extreme conditions that can function as important ecological insurance mechanism, and (iii) the extraction of biochemical products such as beta-carotene or glycerol.

Some ideas to consider

1-Introduction and unified framework approach for aquatic systems

If we accept the world's aquatic ecosystems as a unit in which we can identify ecotones or boundaries separating more or less clearly differentiated communities/systems, two large poles can be identified that support the greatest diversity of aquatic organisms on the planet: the freshwater and marine habitats. There is an evolutionary and salinity difference, but they share homeostasis and stability in the physical environment as a characteristic, as well as a tendency for the community structure to be determined by biological control. On the other hand, there are both thalassic or marine environments and athalassic or continental ones that also have evolutionary and salinity differences, but they share a common feature: they are not physically stable. The fluctuations and unpredictability of environmental variables lead to the selection of communities where only the most tolerant organisms, adapted to

frequent changes, can thrive. So, in my opinion, the target systems could have been not exactly "saline" systems (that include the sea), but astatic systems. In fact, the ms includes three categories of lakes, coastal lagoons, soda lakes and other inland saline lakes (that would include many other). But it would have been perfectly plausible to consider ephemeral or temporary freshwater systems in which, despite maintaining a salinity that changes little around fresh and subsaline, there is no high diversity and there is a set of species very different from those of permanent lakes due to tolerance to desiccation and short hydroperiod (e.g. many branchiopods and amphibians). Many saline lakes, except some permanent, are also temporary and almost all are fluctuating systems. This leads to a wide set of communities and sets of environmental variables, that complete the whole n-dimensional space of factors that the ms depicts in the upper right corner of figure 5.

Answer: We concur that variability of lake level and permanency of a lake is a key environmental parameter. Hence, we highlight this feature in Fig. 1 (permanency is included as symbol shape) and we included it in regression analyses as a potential predictor. However, it did not have a strong impact on our response variables and was not included in the models with the lowest AIC. Nonetheless, permanency and lake level variability play certainly a key role shaping biotic community composition and differentiating lakes among each other, and we therefore followed the suggestion of the reviewer and highlighted that factor in the hypervolume representation in Fig. 5.

Choosing factors or variables that define the limits of these physically controlled systems is nothing but an arbitrary decision. You can choose one of the axes that you consider very relevant, such as salinity, but whichever one you choose, it will be an arbitrary decision that can be justified based on where we think the main "ecotones" are established. Salinity is one of the axes, but fluctuations, degree of permanence (hydroperiod) and some others are possible. Many may be possible and it is a matter of debate to decide which is most appropriate. Let us follow the path of salinity. But, even when choosing salinity, some lines about this unified framework approach for aquatics systems, could be added to the introduction/abstract (i.e. the key of deviation of the traditional freshwater and marine systems that, in the case of marine, is not salinity).

Answer: There are certainly different variables that can be used to categories the lakes. Their astatic nature is an important one (and also included in our study, see response above). However, there are also a few saline lakes with stable salinity and lake levels. Consequently, an astatic nature as selection criterion would have resulted in a different sub-set of water bodies and potentially in slightly different (but equally valid) results. We followed the suggestion of the reviewer and highlight permanency as key feature in the introduction (lines 139; 149) and added a justification of our selection to start of the method section.

Similarly, the formal expression that the n-dimensional space formed by these axes of variation (salinity, degree of eutrophication, size, hydroperiod, etc.) is the central element of study rather than certain individual lakes or lake types. This would better reflect the reality that the systems are rather large regions in space, interconnected and heterogeneous and submitted to wide time scales changes (daily, seasonal and interannual) which provide identity to each system, not certain values of physical variables and a fixed list of species. This is more or less already expressed in the text but is not clearly the central element. The main idea to highlight is that all the diversity accumulated over a wide range of time and space in a connected region should be added together to give a picture closer to reality. This is

important, for these more realistic values of diversity will increase vs alpha-diversity values measurements in just one of the states.

Answer: We adapted your proposition – biodiversity in saline lakes should be measured by at least integrating over time. We therefore now discuss time-integrated alpha diversity (e.g. Line 361). The importance of managing lakes as meta-ecosystem is also highlighted in our management recommendations, a point that has been strengthened in our revision.

2- Selection of systems and criteria.

Once the axis "salinity" has been chosen, the selection of the lakes finally analysed is relevant to the result, so we can discuss it somewhat. In principle it has been carried out by taking the value of 1 g L-1 as the lower threshold, but discarding a large part of the (saline) marine systems (coastal and oceanic waters) although some coastal lagoons are included. So, the criterion "salinity" is mixed as another consideration (i.e. do not include seawater) is overlapping. This criterion is still acceptable but, like other possible ones, it is arbitrary and other decisions could have been made. These could have led to another view of the results. For example: Discarding all the communities of thalassic/marine origin (since the typical communities and species are different from the continental ones even with the same salinity values due to evolutive reasons). (I do not see it as necessary, but it would have been a perfectly plausible decision)

Answer: Yes, natural systems surely are represented by continua and ordering ecosystems in categories or even defining ecosystem borders is always partly arbitrary. Nonetheless, our definition is ecologically sensible as aquatic communities show large changes when salinities changes from freshwater to brackish conditions and likewise when water bodies have a distinct separation from marine waters, and these were our two selection criteria.

- The paradigmatic meeting between the two mentioned types of homeostatic waters occurs in transitional waters on the coasts, estuarine waters, which due to this boundary characteristic and the dynamics of hydrodynamics become waters with a salinity deviated from both large homeostatic poles and are also fluctuating and variable. In this estuarine framework, it is known that works such as Remane & Schlieper (1971) identify an "ecotone" around waters of relatively low salinity (3 to 10) that host a minimum of diversity: a divergent boundary between freshwater and marine systems. Looking for a criterion based on observations similar to this, in which an "ecotone" is identified, the compilation could have moved its limits to e.g. 3-5 g L-1. This decision would have led to considering hyposaline lakes as the beginning of the salinity gradient studied rather than also including subsaline ones (<3).

These two options (which do not necessarily have to be taken if justified) would have consequences on the results presented, such as, for example, the detection of a considerably lower diversity and a relative loss of importance of some services, such as fisheries. So, results and conclusions depend on these decisions on limits, leading to the need to justify or relativize the conclusions under discussion. The reality is that it is difficult and arbitrary to draw boundaries between categories of aquatic systems because, although there are sometimes very marked ecotones, they are actually open communities.

Answer: We have now clarified the section in the methods section that justifies the selection criteria for lake's inclusion in this study. Different selection criteria for e.g. salinity could certainly change our results

slightly. However, we show very clearly how dependent variables (e.g. the number of red-listed species and the provision of individual ecosystem services) changes with salinity and hence potential impacts of more stringent salinity selection criteria can easily be evaluated by the reader. A wider spectrum of lakes comes with the advantage of covering the full range of the ecotone.

In the current version, lakes > 1 g L-1 have been included in the analysis. However, in table S4.1, which describes the characteristics of the lakes to be studied, there is one with only 0.17 g/L (fresh water, L. Epecuen) that should be eliminated of the analysis (or review the value of salinity) and there are 14 with nominal salinity ≤4 with salinity ranges that take the waters to limits close to the lower border of subsaline waters. Figure 3 shows graphically how the "fisheries" service is clearly restricted to this type of waters bordering on the freshwater area and this selection will have also consequences on diversity. So, either reduce the list or mention this in discussion of results.

Answer: Thank you for spotting this mistake. We have corrected the entry to the true value provided by the contributing researcher (170 g L^{-1}). In the analyses, the correct value has been used. The decrease of fisheries with salinity is mentioned in the result and discussion section.

4- Value of ecosystems based on diversity

Do we really have to evaluate systems only by the numerical diversity of already known species? by the number of taxa on a red list without any additional consideration? I know that these are the tools we have to raise well-founded numerical answers, but perhaps in the discussion we should highlight other values beyond that. The reader should see that the analysis is acceptable, but also based on conventional criteria and, at least, open to criticism.

Answer: We very much appreciate this comment – we agree that number of red listed species and the number of ecosystem services are just tools but they only partly capture why we should protect saline lakes. This point was under-represented in our ms and we have improved that point in the revision (see responses below).

First of all, although recognised in the text, and especially in high salinity lakes, aquatic invertebrates and unicellular organisms are crucial explaining biodiversity. But they are almost not addressed in the study (although are widely known by the authors). Besides that, species still to be described by Science are probably in a larger percentage than in marine or freshwater systems due to less research effort. These facts (especially first) could add a lot value to diversity beyond that shown in figure 2 and should be especially highlighted even in the figure caption for hurried readers. This would add to the comment already made about seeing diversity as the sum of a whole series of states.

Answer: We followed the advice and included this point in the figure caption. We initially considered whether we should evaluate the diversity of e.g. planktonic organisms. However, we decided against it in a setting of a meta-analyses as sampling effort (e.g. in terms of covering different lake-level stages) and taxonomic identification skills for these groups can vary and lead to biased results. Hence, we focused on the more robust assessment of red-listed taxa, and we have highlighted our reasoning for this in Line 445.

Also, it is well known that the increase or deviation in salinity values (above that of freshwater or the

sea) leads to a significant decrease in diversity. Should we say that the broad spectrum of saline lakes has a lesser value?

Answer: We agree. Given that ecosystem diversity is a key level of biodiversity in the CBD, which defines diversity of genes, species and ecosystems as crucial considerations, we have now strengthened this point in a section at the end of our result and discussion section.

Perhaps it should be mentioned that equating the value of a system to the known species diversity could be a simplification. This can be the case of hypersaline lakes, as well as other extreme systems on our planet: Extreme environments on the Earth tend to be very little diverse, but they host unique organisms, capable of carrying out processes that are not redundant within the framework of the biosphere and, hence, are of high interest. So, a limited idea of species diversity (as represented in figure 2) is not a complete measure of the value of these ecosystems. Much of these systems have behaved as competitive refuges for highly-tolerant species, guarding very unique and valuable genes in the context of physiological adaptations. Let us not forget that one of the reasons for preserving biodiversity is to maintain a greater degree of resilience of the biosphere in the event of changes. The genes of microorganisms and, sometimes, very halotolerant metazoans (e.g. Artemia spp) guarantee the planet's capacity to adapt to changes. A first-order ecosystem service could be that of constituting gene reservoirs capable of producing and growing in very severe environments.

Answer: This is an interesting point that complements other arguments in our ms, and we have now highlighted it in our discussion.

Another note on diversity would be to consider the different levels of it, especially beta-diversity or habitat diversity. Coming back to the consideration of the multidimensional space that define these systems, a goal of conservation management could be to preserve not only some alpha-diversity linked to a specific coordinate or to a specific space and time, but rather, guaranteeing regional conservation of beta-diversity.

Answer: We agree that beta-diversity is an especially important facet of biodiversity in saline lakes. We now evaluated the rarefaction curve for red-listed species in our analysis (see Fig. 2). We considered this approach as a valuable addition to our analysis.

5-Ecosystem services

According to the summary, there is another pillar supporting the arguments for paying attention to the conservation of the treated systems: the services they provide to society. The identification of ecosystem "services" as a very relevant requirement in the society in which we live, may urge the inclusion of fisheries (and other included in figure 3 and table 1) as something fundamental, but, in my opinion, we must dare to claim the value of the saltiest lakes without fear that the potential services are not so evident.

Answer: We agree (see responses above), but consider the analysis of ecosystem services as an insightful approach.

As Figure 3 shows with colour coding, services can be classified into categories (provisioning, supporting and regulating and cultural). But there is also a prior distinction among them. Pollution is identified as a stressor in the text, and at the same time "wastewater discharges" are considered an ecosystem service and added to a list of values and advantages that lead to its conservation. Although it can be a fact, I think the text should lead the reader to think that the difficult balance between pressures/impacts/stressors and ecosystem services and their regulation is a key for management. Let's say that they are convenient because they are actually being used for the identified services but some sentence should always remember that the basis of their conservation is to intelligently manage those services, since some are, at the same time, clear impacts. However, other services do not cause as much impact or are even provided by the systems simply functioning naturally (e.g. dust retention, carbon sequestration and many cultural and scientific ones). Exposing some of these services only as an advantage or value but not as a possible source of impact can be confusing if not worded appropriately. So, many of the ecosystem services must be clearly regulated in the management process so as not to become a threat, pressure or stressor, in short, to be sustainable.

Answer: Yes, the overuse of most ecosystem services can result in a stressor for the lake's biological communities (e.g. salt mining in saline lakes can be a fundamental issue), and we now caution in a separate paragraph that the use of ecosystem services needs to considers limits for sustainable use (Line 280).

Table 1 and discussion on it. Although it is clear that this is a compilation of current quantified services (e.g. t year-1 km-2…) there are no uses of extraction of products related to non-fish biomass, whether invertebrates or products such as β-carotene or glycerol that can be obtained, as well as a mention in the text of the future potential related to microbial processes, this in part appears in figure 3. The search for innovative new services and this more diffuse service linked to conservation of valuable genes can also be mentioned beyond the current quantified and more widespread ones.

Answer: We have included biochemical products (beta-carotene or glycerol extraction) as potential future ecosystem services.

These reflections could lead to relatively brief changes in actual redaction but I think that could be necessary in the evaluation of the importance of these lakes.

Answer: There is a broad consensus among our co-authors with the raised points and we indeed believe that your comments helped us to substantially refine our submission. It was very pleasing to receive in times when scientific working is often taking place under substantial time pressure, such in depth and constructive criticism in the review process.

Some comments on smaller details (in order of lines)

Line 113. Change to Sustainable

Answer: Spelling mistake corrected.

Line 117. Abstract. See general discussion. Just a suggestion to add some comment not only on "red-listed" diversity but also the consideration that many taxa are not considered in this analysis as

information comes mainly from vertebrates, as well as a brief mention to the uniqueness of the gene pool in the biosphere context especially in the saltiest lakes.

Answer: We have implemented the reviewer's suggestions at many places in the ms. However, the abstract is objected to very tight word-count limitation and we hence decided to not mention this point as it is not linked to our key results.

Line 135 (and table S4.1) review either the limits of the analysis (>1 gL-1), the real value of nominal salinity (0,17 g L-1) of Lake Epecuen (S. America) or eliminate it of the data pool (also species and services etc).

Answer: The value for L. Epecuen in Table S4.1 was incorrect, and we have corrected the entry. As mentioned earlier, we now also present a clear justification for using 1 g L^{-1} as threshold for lake included in our analyses.

Line 138 (and figure 5) Just a suggestion to add the axis of hydroperiod (degree of permanence or frequency of desiccation) to the small graph of multidimensional space top right. It is an ecological sound factor determining the type of aquatic community (tolerance to desiccation) and giving uniqueness to the lakes and list of species, even more than bathymetry or other factors already depicted.

Answer: Variability (e.g. depicted by the permanence of a water) of lake levels and the resulting frequency of desiccation is a key variable; we had already included it in text above the drawing in Fig. 5, and we have improved this point in the graphic display.

Lines 158-160. As already said, when aquatic systems are considered, there are two major sources of diversity and ecosystem services: continental freshwater systems and marine systems (the latest are never mentioned and win in the salt extraction and fish harvest categories). It is difficult to argue that salt lakes provide a comparable level of services or species diversity in a somehow competitive comparison. This is why I believe that the uniqueness of the species and the services they currently or potentially provide should be highlighted, as well as the innovative potential of new services.

Answer: We agree that the conservation and societal value of saline lakes is also resulting from (a) the uniqueness of the habitat types, resulting in large beta-diversity and (b) fundamental supporting services (e.g. impact on micro-climate or the importance of their unique gene pool stated by the reviewer). Hence, we added here a reference to supporting ecosystem services, and addressed these points in a separate section (see above). Further, we have added an analysis of rarefaction curves to highlight the large beta-diversity in saline lakes (Fig. 2).

Lines 165-170. Use these argumentations in discussion of results when both the diversity and the current services are valued.

Answer: We followed this advice – see previous responses.

Line 190 and 201-205. As the assessment of biodiversity in the broadest sense in these systems is a fundamental theme underlying this article, it is clear that an analysis of red lists full of vertebrates, some

of which do not have a critical dependence on water characteristics comparable to aquatic organisms, is insufficient. This fact is acknowledged in this section, but perhaps it should add a recommendation of good practices for managers to assess the distribution of invertebrates. In fact, the addition of aquatic invertebrates to a red list or ascribing them to an IUCN threat category is not comparable to that followed for many vertebrates, but should rather be based on the area of occupancy of the species, i. e. to analyze the IUCN criterion B2, see for example García de Lomas et al (2007 -DOI 10.1007/s10750-017-3322-0). All of this without forgetting to identify unicellular organisms with a future potential to provide new ecosystem services.

Answer: We now address these points in the final section of the paper, where we point out that the urgency to protect saline lakes emerges not only from the number of red listed species and the ecosystem services they are currently provide, but also from the need to protect the diversity of ecosystems (as established by the Convention on Biological Diversity). This is due to large between-system differences especially relevant for saline lakes. Additionally, the point about 'hidden ecosystem services' is included as a key argument in this section.

Line 196. (cited Figures) Figure 2 appears in the pdf as pixelated with low resolution (e.g. species names). Try to put one with a higher resolution that when enlarged on the screen allows you to appreciate more detail.

Answer: We now updated the figure and improved the resolution.

Line 211. The tendency for the diversity to decrease with salinity is well known but, at the same time, as already mentioned, organisms' specialization increases. Physically controlled systems, considered as competitive refuges, but also reservoirs of unique functions, are naturally not highly diverse, but when considering what needs to be conserved, the number of species should not be considered alone.

Answer: The comment is addressed in last manuscript section. See responses above.

Line 219. And the same goes for the size of lakes (or other factors in the n-dimensional space defining types of aquatic systems). It is true that large lakes or permanent lakes can reach a greater number of species, but biodiversity must be analysed in all its aspects. In this case, the level of beta-diversity or habitat diversity is crucial. We should consider the set of aquatic systems as a whole. Considering the global set of species along all the gradients in that multidimensional system and the balance of all those systems (the search for the preservation of beta-diversity) is a desirable goal. The same applies to consider the sum of species throughout a year or series of years in a set of interconnected lakes rather than diversity measured in a punctual state. If we are guided only by the numerical value of their alpha-diversity to prioritize ecosystems conservation, we may be simplifying things and opening a door to lose valuable sets of species. In the current redaction of the ms. the importance of just "diversity" mainly in the sense of number of species in a local site or sample (alpha-div) and the services can lead to forget other valuable elements justifying conservation. It is therefore advisable to convey that what is desirable is the conservation of the entire set of states in the space of environmental gradients, all the resulting communities and the entire range of sustainable and conservation-compatible services, whether current or potential.

Answer: We now refer in the revised ms to the CBD that states explicitly that the diversity of ecosystems is equally important as species diversity.

Lines 235 and 244. Pollution is identified as a stressor and at the same time "wastewater discharges" are considered an ecosystem service and added to a list of values and advantages that lead to its conservation. OK. That's a fact. But I think the text should lead the reader to think that the difficult balance between pressures/impacts/stressors and ecosystem services and their regulation is the key. Some sentence should remember that the basis of their conservation is to intelligently manage those services, since some are, at the same time, possible impacts.

Answer: We now highlight the use of many ecosystem services needs to be balanced with conservation goals.

Line 239 Review "indivudal"; Line 260. arctic instead of artic; Line 292 Review "regulationg"; Line 305 Review "salin"; Line 338 Review "buissnes" and "polical"

Answer: We corrected these spelling mistakes.

Lines 325-327. This fact (complexity of states not only from lake to lake but also within a single lake, see e.g. Garcia et al 1997 doi.org/10.1023/A:1002983723725) is identified as a "difficulty" in managing saline lakes (perhaps ecosystems at a global level). But it is a fundamental reality: it is not a species, nor a lake, nor a single state that society must preserve but these dynamic and heterogeneous processes or functioning. The challenge for the future is to design management schemes and procedures that take this feature into account in an integrative way.

Answer: We adapted the text slightly to make this point explicitly.

Line 410 and others (Methods)
A good effort to gather data from many lakes around the world and to gather the opinions of various experts through targeted questionnaires. At the same time, it is recognized that it is very complex to organize so much and such heterogeneous information. The text of the entire manuscript sometimes loses order and organization perhaps due to this, but in general it is good and I see no objections.

Answer: Thank you for your comments – we are confident that the inclusion of your suggestions has helped us to improve the articulation of our main messages.

Figures and tables

Already mentioned: Figures could be designed to see details better. This is especially necessary for figure 2 that should be included in a much higher resolution. Currently much people try to enlarge figures in a digital platform (tablet, computer…) and this action in the actual pdf copy lead to blurred pixels.

Answer: We improved the resolution of Fig. 2 to ensure the readability of the small text elements.

Figure 3 (comment): I do not understand well "Formal Protection" as a Service… perhaps this could be explained in the text. The fact that a lake or basin is protected by regulations does not make sense to me

whether it is a service that the ecosystem provides to society or vice versa. But I am not used to thinking in services and do not know recent trends defining them. At the same time, it is curious to observe that a set of regulations about the limits of the complete set of services is a service itself.

Answer: Formal protection is not really a service and was not counted in our analysis as an ecosystem service. We nevertheless included it in our analysis the number of red-listed species, because both reflect to some degree the diversity of lakes' natural communities, which is considered as a supporting ecosystem service. We have clarified this in the methods.

Figure 5 (comment): This figure is the core of conclusions and I like it. It summarises well many things that appear somewhat more scattered in the text. It is much more satisfactory outlining the challenges of management in that n-dimensional space, a characteristic that should be highlighted. But, few clues as to how to solve these challenges, I think that some trends or proposals should be written in the text as already pointed out. Perhaps educating the manager in integration and the existence of this complex set of states and factors and the need to see sets in space and time based on long-term monitoring directed to connected systems (which in the graph have been identified as Meta-ecosystems).

Answer: We have modified the n-dimensional space as suggested. We also revised the wording of the management recommendations to account for your comments.

CMG

Reviewer #2 (Remarks to the Author):

Review of NCOMMS-25-03940

The manuscript of Burian et al. is based on the premise that saline lakes are more important for providing biodiversity and ecosystem services than previously acknowledged. While I agree that saline lakes are understudied aquatic ecosystems that deserve more investigation and attention of the scientific community, I find most of the study's results overstated and sometimes even misleading.

Answer: Thank you for your time and efforts to review our manuscript. We have carefully reflected on the raised points of critique, highlighted what improvements we made and clearly expressed our reasoning when we disagree with the reviewer's opinion.

One example is already in the 1st sentence of the manuscript. There, the authors claim that "saline lakes are a diverse group of ecosystems that account for over 45% of the global inland water volume", citing the publication of Messager et al. (2016). Thereby, Burian et al. try to emphasize the global quantitative importance of saline lakes. Upon a closer look, figure 1 the paper of Messager et al. reports 44% of global lakes to be "large salt lakes" and I assume the number cited above refers to this figure. However, 41 of those 44% are represented by the Caspian Sea alone. As a consequence, only 3% of global lakes are salt lakes other than the admittedly huge, but single water body of the Caspian Sea.

Answer: First, we consider this a minor detail. In principle, we are willing to remove this citation if the editor also favors this option.

The citation is factually correct, and the study by Messager et al. (published in Nature Communications) has been cited frequently in a similar way in articles on saline lakes. Although, we can relate to the comment of the reviewer, we think that it is important two consider: (i) some other large saline lakes with substantial volume (e.g. Aral Sea, Lake Texcoco) almost disappeared, because of reasons we cover in our ms. Their disappearance has resulting in enhanced skew toward the Caspian Sea. (ii) The volume of freshwater lakes is also dominated by very few lakes (four lakes contribute to over two thirds of freshwater surface volume). However, a focus on lake volume is to a certain extent arbitrary and we now state both the volume and surface area provided by Messager et al.

Without doubt, saline lakes are extreme environments. Such extreme environments typically have low biodiversity in terms of metazoans, although the authors correctly state that microbial diversity in saline systems can be high. First of all, the low biodiversity of metazoan life in extreme environments is neither a novel finding nor surprising, considering that only few specialized plant and animal taxa can survive under the extreme osmotic stress of saline and hypersaline lakes. If plant and animal diversity is low and saline lakes are admittedly under strong pressure from direct anthropogenic and indirect (climate-change related) stressors, the effect is that a high proportion of the population status of the metazoans in those vulnerable systems is critical, leading to the inclusion of those taxa in the red lists of endangered taxa. However, the study's line of arguments becomes self-contradictory here. As noted above, the authors emphasize the saline lakes' high microbial diversity, thus making saline lakes highly diverse ecosystems. But microbes are not covered by red lists of threatened species. Nevertheless, the authors argue that the high proportion of threatened (red list) animal species found in saline lakes is evidence for the – as they argue overlooked - high biodiversity of saline lakes. As such, this line of

reasoning does not make sense and the study states in line 193 that the saline lakes in their study hosted a median number of only three threatened species. Although I do not dispute the value of conserving saline inland waters, these low numbers cannot be considered proof for the claim that saline lakes harbour a large number of threatened aquatic biota, as made by the manuscript.

Answer: We do not agree that our line of argument is self-contradictory. Our point is that not only aquatic species but also terrestrial species, whose presence critically relies on the existence of saline lakes, should be taken into consideration when evaluating the conservation value of saline lakes based on red-listed species. If one does so, there is a much larger number of red-listed species relying on saline lakes than commonly perceived, although aquatic metazoan diversity is low in saline lakes.

Further, it was never out intention to write a comparative study and evaluate whether saline lakes support more red-listed species or ecosystem services (see below) than freshwaters. Instead, our main message is that there are many more red-listed species in saline lakes than commonly perceived, because there are also many terrestrial species that critically rely on saline lake ecosystems. This important message is clearly articulated in several places in the ms (e.g. Abstract: Line 124, Result and discussion Line 200, Conclusion Line 405). It is not a very fruitful discussion to debate whether a median value of three red-listed species per lake is a high number (though it is in our opinion not self-evident that freshwater lakes with a median size of 13.8 km^2 would have a substantially higher count). Important is that three red-listed species is clearly a large enough number to justify their protection – which is urgently required given the substantial pressures that endanger the existence of many saline lakes.

Finally, the reviewer touches on a point that was also made by reviewer 1 – that an important element of the value of saline lakes is the diversity at the ecosystem level. We fully agree with this statement, now integrated more clearly in our ms.

Another key claim of the paper is that saline lakes provide particularly relevant (cultural and economic) ecosystem services. Looking at the list of investigated ecosystem services in this study however, all of them (with the exception of salt mining) are ecosystems services that are provided by all kinds of lakes and not specific to saline lakes at all. While I will not dispute that conserving the world's lakes and their ecosystem services (which includes saline lakes) is of utmost importance, I do not see why the saline lakes should deserve this particular emphasis over lakes in general. I would even presume that non-saline lakes could potentially provide even more ecosystem services than saline ones, in particular services related to drinking water and fisheries (as fish densities are much higher in non-saline lakes, as stated in line 264).

Answer: It is a misconception that we claim that saline lakes should get more attention than freshwater lakes. In reality, saline lakes receive much less attention by decision makers than freshwater lakes, and they are often perceived to be of no use to humanity. Our analysis challenges this view, and notes the surprising finding that the number of provided ecosystem services does not decrease with salinity.
It is clearly not the target of our manuscript to compare freshwater and saline lakes. We are surprised that the reviewer implies that we do and we carefully reviewed the text of our ms to ensure that we do not create this impression. Comparisons of ecosystem services between freshwater and saline lakes are probably more meaningful than tallying red-listed species in different habitat types. E.g., it was interesting that fish harvests in those saline lakes that provide the services are very high compared to freshwater lakes – most likely because saline lakes are often naturally eutrophic. Nonetheless, it was

again not our main intention to compare freshwater and saline lakes, but to highlight that the contributions of saline lakes to human society are much larger than commonly perceived. We are convinced that this is an important finding.

Last but not least, not a single of the proposed “best-practice recommendations for sustainable management of saline lakes” (line 128) is exclusive to saline lake systems. Most of them – while not incorrect – are very generic and probably applicable to inland waters in general.

Answer: Thank you for the comment – we agree that it would be better to highlight that our suggestions are of particular importance for saline lakes. For example, catchment-wide water management is key for freshwater lakes to manage nutrient and turbidity levels. Nonetheless, it is arguably more important in saline lakes, where a failure to manage water balances will lead to the disappearance of aquatic habitats. We have revised this section to better articulate such differences.

REVIEWER COMMENTS

Reviewer #1 (Remarks to the Author):

Remarks to authors
After reading the revised manuscript, I have noticed that my review work has been appreciated by the authors (for which I am sincerely grateful, as preparing those recommendations took quite some time and was based on personal ideas developed over years—something rarely acknowledged). Most of the ideas I had previously noted as missing from the text have now been incorporated into specific lines or paragraphs. From this perspective, and also appreciating the new organisation of the section on examples of good practices and the changes made to Figure 2, to mention just one example, the manuscript has improved.

Response: Thank you again for the many valuable suggestions and constructive comments. We are well aware that the time committed by the reviewer clearly exceeded the standard expectations and we are grateful for the detailed feedback from which our article substantially profited.

However, in my opinion, these aspects could have been developed a little further and, above all, the central conceptual framework or main message of the potential article has not changed substantially.

Response: We concur that there are limitations to the ecosystem service concept as well as red-listed-species based assessments, and that these limitations need to be stated clearly. We followed the advice of the reviewer in our revision and aimed to not only acknowledge these issues but turn their discussion into a core part of the ms. Key implemented changes include:

- Abstract: We now highlight the importance of ecosystem diversity (i.e. beta diversity) complementing counts of threatened species. Further, we now emphasise that a number of important ecosystem services are difficult to quantify.
- Last paragraph of Introduction: We state the need to account for limitations of quantitative ecosystem service assessments and explain that the evaluation of such limitations is a core part of our evaluations (line number 185-188).
- Conservation value section of Results and Discussion: We added a paragraph to underline the importance of genetic diversity in saline lakes. Further, we clearly state now that the conservation value of saline lakes emerges from (i) strong aquatic-terrestrial coupling that largely extends the number of threatened species, (ii) the large diversity at the ecosystem level and (iii) the unique gene sets that are only encountered in saline lakes (line number 234-243).
- Subtitling a section of the Results and Discussion as ‘The truly hidden ecosystem services of saline lakes’. We use this part of the paper to highlight key supporting and regulating ecosystem services that are difficult to quantify but of essential importance for human well-being and potential ecosystem services that have not been materialised but can be of substantial importance in the future. In this section, we also clearly explain that the functional importance of saline lakes emerges from the sum of quantifiable services, difficult-to-quantify supporting and regulating ES and evaluation of future potential for ES provisioning (line number 295-342).

We believe that implementing these changes and following the recommendation of the reviewer to integrate conceptual critique in core parts of our evaluations has helped to broaden the scope of our work and thereby substantially strengthened our manuscript.

The manuscript continues to insist on a message that assumes that the conservation of systems is justified by their diversity values (quantified solely through larger or vertebrate species, with particular attention to red lists) and by the ecosystem services they provide. As I mentioned in my first review, there are two large groups of ecosystems that are better studied, more stable, and at the same time much more diverse, that are used more intensively by humankind globally. In fact, they are vital for oxygen supply, gas exchange and other biological and physicochemical processes—namely, freshwater and marine environments—so the central argument loses strength as a key justification.

Response: We agree with the reviewer that a focus on quantifiable ecosystem services is linked to limitations. Hence, we have now emphasized these limitations of applied concepts and added a qualitative synthesis of non or less-well quantifiable services.
Further, we agree that marine systems show much larger contributions to global ecosystem service provisions. But given that microbial activities in saline lakes can be substantially higher than in freshwaters, we would argue that global rates for a number of supporting ES (gas exchange) are similar in freshwater and saline lakes. We have no added this as a minor point to our discussion (line 318-322). However, a global comparison of the functional roles of freshwater, marine and saline lakes is well beyond the scope and not the intent of this study. Instead, our central argument is that saline lakes are often perceived as just a part of the barren drylands that frequently surround them. We believe that our synthesis of quantifiable as well as of the non-quantifiable ecosystem services, which we now added, clearly refute this notion. We have highlighted this point clearly at the end of the section entitled 'The hidden ecosystem services of saline lakes' (lines 333-342).

It is a fact that these other two types of environments exhibit greater diversity, even if, as is now discussed (and was less so in the first version), global biodiversity is considered taking into account genes, habitat-level or beta-diversity, and a more integrated vision across longer time scales, not just on point inventories.

Response: Thank you for this comment. However, we did not aim to compare at any point in the manuscript the global diversity supported by saline lakes with those of freshwater and marine ecosystems. Further, we believe that comparisons between ecosystem types -if they were made – should at least partly rely on area-based measures. At an area-based scale – and we believe based on the first revision round that we are here in principle agreement with the reviewer – diversity in saline lakes is considerable for the following reasoning:

(i) strong aquatic-terrestrial coupling that substantially extends the number of threatened species that depend on saline lakes;
(ii) the large diversity at the ecosystem level (Fig. 2B) due to complex environmental hypervolumes (Fig. 5);
(iii) the unique gene sets that are only encountered in saline lakes.

We have now clarified this reasoning in the manuscript at the end of the section 'Threatened species and the conservation value of saline lakes (lines 333-342). These points have now been much clarified thanks to the exchange with the reviewer, and we believe they capture very essential reasons why should intensify our efforts to conserve saline lake ecosystems.

Regarding ecosystem services, it is clear that fishing (one of the first services that comes to mind for the common responsible for benefits when aquatic systems are mentioned) is here concentrated in lakes in the hyposaline and subsaline margins, rather than across the broader salinity gradient of continental saline lakes. Other services expressed in Figure 3 and later in Table 1, such as wastewater discharge, tend to be provided more massively and are more easily managed by seas and large lakes, without posing the intolerable pressure or impact found in many saline lakes of smaller volume, which would need careful analysis and management. Research, recreational value, and the presence of species of interest are not a particular strong point for this type of systems either, since many of these services in freshwater and marine systems represent higher and mostly far superior values. Even in the case of salt extraction, marine salt pans are of much greater importance. Therefore, the very conception of the article (namely, presenting these two pillars -diversity and services- as solid arguments for conservation) still does not seem to me the most satisfactory approach to demonstrate their value, at least not the value they deserve.

Response: We agree with the reviewer that supporting and regulating ecosystem services that are difficult to quantify might be even of greater importance than provisioning services. Hence, we have now placed a much greater emphasis on such services (see responses above).

Nonetheless, we would caution from disregarding provisioning ecosystem services in saline lakes. Average areal fish yields, for example, are in those saline lakes that provide fish much higher than in freshwater and marine systems. Further, salt extraction is targeting different elements in saline lakes and marine system and hence fulfils a different commercial purpose. The touristic value of saline lakes, is just as that of freshwater lakes, highly case-specific. Notable examples that provide high touristic value are Lake Nakuru, which is one of only three national parks in Kenya that generate net positive revenue, and the Dead Sea, one of the main natural tourist attractions in Israel. Hence, we aimed for a balanced assessment in our revision that considers both, quantifiable provisioning and cultural as well as more elusive regulating and supporting ecosystem services.

I believe I made it clear in my first review that I am fully convinced of the need to conserve these systems and that I consider them extremely valuable. It is true that the idea I mentioned about the uniqueness of the genes of highly tolerant organisms, which contribute resilience to our biosphere, has been incorporated into a paragraph in the new version, which I greatly welcome. However, assuming as the foundation of everything, that ecosystems are valuable only, or just to the extent that they provide numerous quantifiable services to humankind or host a considerable number of species already included on red lists, seems to me, personally, a kind of concession to a modern social consensus and prevailing evaluation frameworks that, sometimes, are susceptible to some criticism.

Response: We would argue that biodiversity, reflecting the intrinsic value of ecosystems and the provisioning of ecosystem services, reflecting their functional importance, represent two core elements why we should conserve saline lakes (and ecosystems in general). We agree with essentially all core points raised by the reviewer - i.e. that biodiversity assessments also need to encompass genetic and ecosystem levels and that evaluations of quantifiable ecosystem services are limited and many difficult-to-quantify services also need to be considered. In order to strengthen these points in the manuscript, we have implemented the changes listed in our second response to the reviewer.

It might be worthwhile to add a brief critical note on this widespread perspective, even daring to touch upon a somewhat romantic notion related to their intrinsic values by using, for instance, the uniqueness of those genes and processes. Personally, I always find it somewhat strange to have to justify the conservation of ecosystems and species based on their participation in "beneficial" processes, and I sometimes feel a little outside the established system. In that sense, another possible approach would be to present the fraction of diversity related to vertebrates, that is measurable and easy to quantify, and the services they may provide as an added value rather than as the main argument. This could even be reflected in the title (if it were up to me, I would leave it as "The Underestimated Conservation Value of Saline Lakes"). But now the title in fact, makes services the central foundation of the manuscript. In any case, as I have hinted, I feel that, after a long time studying aquatic systems, I have observed changes in how society as a whole perceives the value of ecosystems, and at times I have not been able to adapt fully to some of these changes. Thus, there is a need for the values to be easily quantifiable and readily integrated into economic or social models by a sector of society that does not perceive the "invisible", as already briefly mentioned in the new version.

Response: We concur that there is a tendency, if not pressure, to frame the need to conserve nature based on utilitarian principles. Our manuscript does incorporate biodiversity and the provisioning of ES both intrinsic and functional arguments for saline lake conservation. However, both elements would be captured in the title "The Underestimated Conservation Value of Saline Lakes". Hence, we followed the recommendation of the reviewer and adapted the title with small modifications (specifically, we kept overlooked instead of underestimated, but if the editor would recommend us to us underestimated instead, we have no concerns with making this change).

I am fully aware that this viewpoint of just a person may be legitimately criticised by the authors from the 58 prestigious institutions that have signed the manuscript. I also know it may or may not be shared by the editors. Therefore, I do not consider myself an infallible judge.

For that reason, I will not issue a definitive verdict indicating whether I accept or reject the manuscript. However, my personal opinion is that it can at least be improved (it remains open to revision), and I believe its focus and structure could be reconsidered to the point of turning it, from my perspective, into an almost entirely new article. So I will qualify it as "need major revision" again.

Response: Thank you again for the many thoughtful and helpful suggestions!

Carlos M García
University of Cádiz
Spain

Reviewer #2 (Remarks to the Author):

I appreciate the authors' detailed response to the comments I had raised on an earlier version of

their manuscript. They have clearly amended and improved the style of their manuscript. I do not find anything technically wrong with their analysis. Still, the sole purpose of this manuscript is to raise awareness on the understudied saline inland waters. While this is a valid opinion, there is not much scientific novelty in this paper that would provide significant advancement to the field.

Response: Thank you for again reviewing our manuscript. We are glad to hear that the revisions were satisfactory and our improved manuscript is technically robust. Regarding the evaluation of scientific novelty, we acknowledge that this is an inherently subjective process where professional opinions may diverge. However, we maintain that this study offers two major, unprecedented contribution to the research field: (1) To our knowledge, this is the first comprehensive global-scale synthesis of ecosystem services provided by saline lakes. (2) We assembled a “tree of threatened life” supported by these ecosystems. This analysis explicitly highlights the crucial, yet often overlooked, role of aquatic-terrestrial coupling in sustaining biodiversity. We believe these elements represent notable additions to the field, providing a systematic, global perspective on these critically threatened ecosystems.

Reviewer #3 (Remarks to the Author):

I have read this ms and, at the editor's request, I focused on the expert judgment aspects. The paper is well written. Expert judgment in this case is not forecasting values or quantifying uncertainty; it is a simple preference survey. The experts are identified as co-authors. I see no red flags or causes for concern here.

Response: Thank you for your expert opinion and the time invested in reviewing this manuscript.

Two minor editorial remarks: (1) in the abstract there ae 84 saline lakes, but line 227 mentions 85 saline lakes. (2) in line 292 "a" should probably be "are".

Response: We have corrected these two editorial points in our new revision.
