# Supplementary material for: The overlooked conservation values of saline lakes
Source: Nat Commun. 2026 Sep 3;17:9456. doi: 10.1038/s41467-026-76684-0 (PMC13542260; doi:10.1038/s41467-026-76684-0)
Supplement: Supplementary file 4 [file 41467_2026_76684_MOESM3_ESM.pdf]

Description of Additional Supplementary Files

File Name: Supplementary Data 1
Description: Environmental data
The environmental conditions of the included lakes

File Name: Supplementary Data 2
Description: Ecosystem services
The identity of ecosystem services that are provided by each of the included lakes

File Name: Supplementary Data 3
Description: Red-listed species richness
The number of red-listed species per lake

File Name: Supplementary Data 4
Description: Red-listed species identity
The identity of red-listed species recorded fore each lake

File Name: Supplementary Data 5
Description: Template of the questionnaire
The template of the questionnaire used to collect the raw data

File Name: Supplementary Code 1
Description: Annotated R code
