# Supplementary material for: The overlooked conservation values of saline lakes
Source: Nat Commun. 2026 Sep 3;17:9456. doi: 10.1038/s41467-026-76684-0 (PMC13542260; doi:10.1038/s41467-026-76684-0)
Supplement: Supplementary file 5 [file 41467_2026_76684_MOESM8_ESM.pdf]

The template of the questionnaire

General Information

The general criterion for the lake to be included into the survey is average salinity above 0.5-1 g L^{-1} and size of the lake above 0.05 km^2. For clarification, examples of answers are provided. Lines in bold are compulsory questions.

**Lake name**	**e.g. Lake Shira**
**Country**	**e.g. Russia**
**Coordinates**	**e.g. 54°30′ N 90°12′ E**
**Lake Size**	**km^2**
**Volume**	**m^3**
**Maximal depth**	**m**
**Average depth**	**m**
**Salinity, average**	**g/l**
**Chemical type of the lake (based on dominating ions)**	**e.g. carbonate (soda), sodium sulfate, magnesium sulfate, chloride, etc.**
**Lake origin**	**e.g. terminal lake in endorheic basin, karst lake, coastal lake, crater lake, man-made pit lake, etc.**
**Observed fluctuations of salinity in past 50 years**	**Yes or No**
**Magnitude of recent (during last 50 years) salinity fluctuations**	**e.g. 2-25 g/l**
Desiccation event in last 50 years	Yes or No<br>If yes, number of events

Ecosystem characteristics

Maximal, minimal and typical Secchi depths during growing season	m
Mixing regime	e.g. shallow-mixed, monomictic, dimictic, polymictic, meromictic, etc.
Average and maximum summer (growing season) chlorophyll concentrations	mg/m^2
Dominant phytoplankton species	Latin names
Dominant zooplankton species	Latin names
**Fish presence**	**Yes or No**
Dominant fish species	Latin names

Estimate of total species richness (at least for vertebrates and invertebrates over >1mm in body size. For smaller size organisms like nematodes, algae or bacteria we usually do not have a reliable count. However, if you have reliable estimates for smaller organisms, you can also provide them).	e.g. invertebrates - 20 species, vertebrates - 5 species, terrestrial vertebrate species depending on the lake (waterfowl, etc.) - 3 species
**Lake has associated wetland that directly dependent on the existence of the lake**	**Yes or No**
Approximate area of the wetland	km^2

Conservation Status

**Part of Ramsar Site**	**Yes or No**<br>**If Yes, name of Ramsar Site**
**Protected area (national or regional) status**	**Yes or No**<br>**If Yes, name status**
**Presence of endangered species according to national or international law (e.g. IUCN red list)**	**Yes or No**
List of endangered species (if applicable)	Latin names
Presence of Endemic Species	Yes or No
List of Endemic Species (if applicable)	Latin names

Ecosystem services status

**Lake water is used for economic purposes**	**Yes or No**
Type of water usage	e.g. agriculture, aquaculture, desalination, drinking purposes, etc.
Amount of water used (if available separate estimates for different uses)	for each purpose, m^3 per year

**Water from the closed basin is withdrawn for economic purposes**	**Yes or No**
Type of water withdraw	e.g. agriculture, aquaculture, desalination, drinking purposes, etc.
Amount of water used (if available separate estimates for different uses)	for each purpose, m^3 per year

**Lake or associated wetland is used for the discharge of wastewater**	**Yes or No**
Type of wastewater	e.g. industry, agriculture, cattle, domestic, etc.
**The wastewater is properly treated before discharge**	**Yes or No**
The amount of wastewater discharged (if available) (approximate values)	m^3 per year or wastewater from which no of inhabitants

**Lake shore is populated**	**Yes or No**
Population of cities of villages located at the shore of the lake	number of people

**Lake is used for commercial fishery**	**Yes or No**
**Lake was stocked with non-native fish species**	**Yes or No**
Type of fishery	natural or aquaculture
Estimate of commercial fishery yield (if available) (approximate values)	tons per year

**Lake or associated wetlands are used to harvest other biological resources**	**Yes or No**
Type of resources	e.g. Artemia, Gammarus, algae biomass, higher plants biomass, etc.
Estimate of harvested yield of resources (if available) (approximate values)	tons per year

**Lakes is used for commercial extraction of salts, minerals or muds**	**Yes or No**
Type of extracted resource	e.g. salts, muds, etc.
Estimate of extracted resource (if available, if several minerals or resources – for each resource separate number) (approximate values)	tons per year

**Lakes is used as recreational site**	**Yes or No**
Type of recreation	e.g. camping, hotel, visit center, spa resort, etc.
Intensity of recreation	seasonally or regularly
Origin of tourists	local, regional or international

Estimate of number of visitors (if available) (approximate values)	people per year
Estimate of the average duration of the visit	days

**Lake is used as valuable science research object**	**Yes or No**
**There are at least 20 papers in Web of Science Core Collection searched with “Lake Name”**	**Yes or No**
The distance to the closest large educational or science institution (university, academic institute)	km<br>name of the university or institute
**The lake is used for teaching course and/or There is a research field station at the lake**	**Yes or No**

**Lake provides cultural ecosystem services**	**Yes or No**
Type of cultural ecosystem services	Select appropriate characteristics of the lake:<br>- resonant in terms of culture or heritage,<br>- enable aesthetic experiences,<br>- have symbolic meaning,<br>- have sacred or religious meaning,<br>- etc.

Other ecosystem services provided by the lake	Please add information on any other ecosystem service that is not covered by previous questions (e.g. major effect on local microclimate, development of biotechnological patents based native microbial taxa, etc.)<br><br>To be sure that you covered all important ecosystem services you can check International Classification of Ecosystem Services available here https://cices.eu/content/uploads/sites/8/2018/03/Finalised-V5.1_18032018.xlsx

**Please, select and rank the three most important ecosystem services of the lake, subjective judgment**	**- Provision of water<br>- Provision of biological resources<br>- Provision of mineral resources<br>- Regulation and maintenance of water quality and/or ecosystem health<br>- Regulation and maintenance of local weather/climate<br>- Recreational services<br>- Cultural services<br>- Educational/scientific services<br>- Others (please specify)**

Current and potential threats to the lake

**Does lake experience water withdrawal**	**Yes or No<br>If yes: withdrawn % of total runoff in the catchment**
**The climate change is expected to have a severe impact on the lake's provision of ecosystem services**	**Yes or No**
**What are the main impacts of climate change on the lake**	**e.g. reduced runoff, increased evaporation, water temperature increase, effects of wind, increased climate variability, altered stratification patterns, etc.**
**There are invasive or introduced species in the lake**	**Yes or No**
What are the main land-uses in the catchment (if possible provide % of land cover)?	e.g. industry 5%, agriculture 70%, human settlements 10%, intact ecosystems 15%, etc.
**Please, select and rank the three most important threats to the lake, subjective judgment**	**- land use change and intensity<br>- pollution<br>- climate change<br>- invasive species<br>- overexploitation (e.g. fisheries)<br>- water abstraction<br>- others (please specify)**
